# The Tsetse Fly Displays an Attenuated Immune Response to Its Secondary Symbiont, *Sodalis glossinidius*

**DOI:** 10.3389/fmicb.2019.01650

**Published:** 2019-07-24

**Authors:** Katrien Trappeniers, Irina Matetovici, Jan Van Den Abbeele, Linda De Vooght

**Affiliations:** Department of Biomedical Sciences, Institute of Tropical Medicine Antwerp, Antwerp, Belgium

**Keywords:** *Glossina*, *Sodalis glossinidius*, host-symbiont crosstalk, immune interaction, transcriptomics

## Abstract

*Sodalis glossinidius*, a vertically transmitted facultative symbiont of the tsetse fly, is a bacterium in the early/intermediate state of its transition toward symbiosis, representing an important model for investigating how the insect host immune defense response is regulated to allow endosymbionts to establish a chronic infection within their hosts without being eliminated. In this study, we report on the establishment of a tsetse fly line devoid of *S. glossinidius* only, allowing us to experimentally investigate (i) the complex immunological interactions between a single bacterial species and its host, (ii) how the symbiont population is kept under control, and (iii) the impact of the symbiont on the vector competence of the tsetse fly to transmit the sleeping sickness parasite. Comparative transcriptome analysis showed no difference in the expression of genes involved in innate immune processes between symbiont-harboring (*Gmm*^*Sod+*^) and *S. glossinidius*-free (*Gmm*^*Sod*–^) flies. Re-exposure of (*Gmm*^*Sod*–^) flies to the endosymbiotic bacterium resulted in a moderate immune response, whereas exposure to pathogenic *E. coli* or to a close non-insect associated relative of *S. glossinidius*, i.e., *S. praecaptivus*, resulted in full immune activation. We also showed that *S. glossinidius* densities are not affected by experimental activation or suppression of the host immune system, indicating that *S. glossinidius* is resistant to mounted immune attacks and that the host immune system does not play a major role in controlling *S. glossinidius* proliferation. Finally, we demonstrate that the absence or presence of *S. glossinidius* in the tsetse fly does not alter its capacity to mount an immune response to pathogens nor does it affect the fly’s susceptibility toward trypanosome infection.

## Introduction

Bacterial endosymbiosis in insects is a diverse and ubiquitous phenomenon in nature that has been shown to affect different aspects of insect physiology and is recognized to be a key driver of evolutionary novelty and complexity (Moran, 2006). One important physiological aspect that can be affected by the presence of symbionts is the host immune system (Gross et al., 2009; Hooper et al., 2012). Unlike vertebrates, which are provided with an innate immunity coupled to a specific adaptive immunity to combat infection, insects depend solely on innate defense reactions comprised of cellular and humoral responses together with physical barriers. The cellular immune system is based on the activity of hemocytes involved in phagocytosis, encapsulation, and nodulation of pathogenic microorganisms. The humoral response is based on conserved signaling pathways leading to the release of effectors, including AMPs. These defense reactions are triggered by the recognition of microbe-associated molecular patterns (MAMPs) through pattern recognition receptors (PRRs) and result in the activation of signaling pathways such as the Toll-, Imd-, and Janus kinase/signal transducers and activators of transcription (JAK/STAT)- pathway (Lemaitre and Hoffmann, 2007).

The alimentary tract of insects continuously faces challenges with various microorganisms including commensals, mutualists, and opportunistic microbes or pathogens. Maintaining a long-term symbiotic relationship thus requires the host to respond differently to symbiotic and pathogenic organisms. How host immune defense mechanisms are regulated to allow endosymbionts to establish chronic infections of their hosts without being eliminated can have important implications for host life history traits. On the one hand, symbiont presence can prime the host immune system (Kambris et al., 2009; Rancès et al., 2012) thereby making it less vulnerable to pathogens (Wong et al., 2011; Rancès et al., 2012). Indeed, as there is a functional overlap in the antibacterial, antiparasitic, and antiviral innate immune responses of insect vectors, the microbiome can be a driving force for alterations in the host defenses that also affect establishment and maintenance of pathogens. Recent studies have demonstrated that the natural microbiome significantly contributes to determining the vector competence of blood feeding insect hosts to medically important pathogens (Glaser and Meola, 2010; Cirimotich et al., 2011). For example, both *Anopheles* vectors of human malaria and *Aedes* vectors of dengue fever have been shown to harbor a gut microbiome that stimulate the production of basal levels of immune effector molecules that control the proliferation of the bacterial populations, but also inhibit pathogen development (Dong et al., 2009; Ramirez and Dimopoulos, 2010). On the other end of the spectrum, if host immune responses triggered by the presence of microorganisms pose costs to, or limit the establishment and maintenance of other beneficial symbionts, symbiosis may select for a broadly reduced immune response to facilitate beneficial symbiont maintenance (Laughton et al., 2016). Some studies suggest that pea aphids have reduced their immune repertoire to facilitate its association with beneficial symbionts such as *Buchnera* (Gerardo et al., 2010). While this strategy profits honest, beneficial symbionts (Haine, 2008), it can leave hosts more susceptible to pathogens.

Tsetse flies (*Glossina* spp.) are medically and veterinary important vectors that transmit *Trypanosoma* spp. parasites responsible for human sleeping sickness and animal African trypanosomiasis. In comparison to insects that feed on multiple diets, tsetse flies are colonized by only a small number of symbiotic microorganisms, reflective of their strict hematophagous lifestyle, making them a useful model system for studying host-symbiont-pathogen interactions. The gut microbiome of adult tsetse flies is dominated by only two symbiotic microorganisms, both members of the family *Enterobacteriaceae*: the anciently associated obligate mutualist *Wigglesworthia glossinidia* and the more recently established facultative symbiont *Sodalis glossinidius*. A third facultative endosymbiont, that occurs in some natural tsetse fly populations is an α-Proteobacterium of the genus *Wolbachia* (O’Neill et al., 1993). *W. glossinidia* is found intracellularly in differentiated epithelial cells (bacteriocytes) which form an organ (bacteriome) in the anterior gut. This obligate symbiont has been shown to be essential for female fecundity and for the development of a well-functioning immune system (Pais et al., 2008; Weiss et al., 2011). In contrast to *W. glossinidia*, our knowledge on the role of *S. glossinidius* in relation to tsetse immunity is still very limited. This bacterium displays a wide tissue-tropism and appears to be present in all lab-colony flies, whereas a varying prevalence (0-65 %) has been observed in wild tsetse flies (Cheng et al., 2000; Geiger et al., 2009). So far, no specific functional contributions toward tsetse biology are identified. In fact, little is known about the biological impact of *S. glossinidius* on the tsetse fly’s physiology and how the symbiont population is kept under control. Although the presence of *S. glossinidius*-specific genotypes has been linked to an increased susceptibility of some tsetse fly species for trypanosome transmission (Geiger et al., 2007; Farikou et al., 2010), its actual role in the ability of tsetse flies to acquire and transmit the parasite still remains controversial (Geiger et al., 2005; Channumsin et al., 2018; Tagueu et al., 2018). It has been very challenging to study the physiological roles of tsetse symbionts as antibiotic treatment of fertile flies often results in the elimination of the coexisting essential *W. glossinidia* symbiont, which results in host sterility, making it difficult to generate fly lines devoid of a specific symbiont. In this study, we tested different treatments of the prokaryote-specific antibiotic streptozotocin and managed to establish a *S. glossinidius*-free (*Gmm*^*Sod*^***^–^***) tsetse fly colony. This was obtained by crossing the *Gmm*^*Sod*^***^–^*** offspring of pregnant female flies without affecting tsetse’s other bacterial endosymbionts. The availability of this *Gmm*^*Sod*^***^–^*** fly colony allowed us to compare host immunological parameters between *S. glossinidius*-infected and uninfected flies with identical genetic backgrounds.

First, we performed an RNA-sequencing (RNA-seq) comparative transcriptome analysis of *S. glossinidius*-harboring (*Gmm*^*Sod+*^) and *S. glossinidius*-free (*Gmm*^*Sod*^***^–^***) flies to obtain a global picture of the genes that are differentially expressed in response to the symbiont’s presence. Next, we examined the immune responses in *Gmm*^*Sod*^***^–^*** flies that were exposed to cultured *S. glossinidius* or *E. coli* using RNA-seq and quantitative real-time PCR (qRT-PCR). In-depth analysis of genes involved in immunity demonstrated a moderate immune response elicited by *S. glossinidius*. Otherwise, challenge with exogenous *E. coli* resulted in full immune activation, showing the ability of *S. glossinidius* to induce a weaker immune response than pathogenic bacteria and suggesting the existence of a mechanism allowing immune tolerance of this gut symbiont. We next monitored the effects of attenuating or activating tsetse immunity on the *S. glossinidius* densities in the fly. RNA interference (RNAi)-mediated immune suppression did not affect the *S. glossinidius* population, nor did experimental activation of the Imd-pathway, the main immune signaling pathway in the insect’s response to Gram-negative bacteria (Lemaitre and Hoffmann, 2007). These results indicate that *S. glossinidius* is not susceptible to the tsetse immune responses and that the humoral immune pathway does not play a major role in controlling *S. glossinidius* proliferation. Finally, we report on the impact of *S. glossinidius* on the tsetse fly vector competence for trypanosomes (*Trypanosoma brucei* and *T. congolense*).

## Materials and Methods

### Tsetse Flies and Bacteria

In all experiments, *Glossina morsitans morsitans* (*Gmm*) flies from the colony of the Institute of Tropical Medicine (ITM, Antwerp, Belgium) were used. Experimental flies were fed 3 days/week with commercially available defibrinated horse blood using an artificial membrane system and maintained in standardized environmental conditions of 26°C and 70% relative humidity. *S. glossinidius*-free (*Gmm*^*Sod*^***^–^***) flies were generated using the antibiotic streptozotocin (Sigma-Aldrich). Intrathoracic microinjection (34′ gauge Hamilton syringe) with streptozotocin (dose indicated in Supplementary Additional Files 1, 2) was performed in teneral flies that were first briefly cold-shock anesthetized. After injection, flies received streptozotocin-supplemented or regular blood meals every 48 h. The *per os* treatment consisted of three or continuous streptozotocin-supplemented blood meals (dose indicated in Supplementary Additional Files 1, 2). To establish a *Gmm*^*Sod*–^ colony, treated female flies were mated 3–4 days post-eclosion and pupae were allowed to hatch. The *Gmm*^*Sod*–^ progeny did not receive any antibiotic treatment and flies were further maintained in standardized climatic conditions on a normal feeding regime.

*Sodalis glossinidius* bacteria were grown on insect medium packed with horse blood cells at 26°C under micro-aerophilic conditions as described by Matthew et al. (2005). *Sodalis praecaptivus* were grown on insect medium packed with horse blood cells at 37°C as described by Chari et al. (2015). The *Escherichia coli* strain used in this study was obtained from the OneShot^®^TOP10 kit (Invitrogen) related to the DH10B^TM^ strain and was grown in liquid Luria-Broth (LB) medium or on 1% bacto-agar LB plates at 37°C.

Bacterial exposure in tsetse flies was performed on day 8 post-eclosion by intrathoracic microinjection (34′ gauge Hamilton syringe) of 1 μl with 10^6^ CFU alive *Sodalis glossinidius* (unless mentioned otherwise), 10^6^ CFU alive *Sodalis praecaptivus*, or the non-lethal 10^5^ CFU alive *E. coli*. Bacteria were collected by centrifugation at 5,000 × *g* for 5 min and resuspended in sterile saline. On the same day, flies received a blood meal supplemented with 10^6^ CFU live *S. glossinidius* or 10^5^ CFU live *E. coli* per 20 μl blood, the average amount one fly ingests during feeding (Langley, 1966; Moloo, 1971). The blood serum was first heat-inactivated for 30 min. at 56°C and only fully engorged flies were maintained. Samples were taken 48 h after challenge, which allowed the flies to completely digest the blood meal prior to RNA extraction.

### RNA-Sequencing

Total RNA was isolated from abdomens of 10-day old *Gmm*^*Sod*+^ and *Gmm*^*Sod*–^ flies and 48 h after exposure for the *Gmm*^*Sod*^***^–^****^/Sod+^, Gmm^*Sod*^****^–^****^/Ecoli^*^+^, and *Gmm*^*Sod*–/saline^ groups using the PureLink^®^ RNA mini kit (Ambion) and DNaseI-treated (Ambion). RNA quality was validated using the RNA6000 Nano chip kit on a 21000 Bioanalyzer (Agilent). RNA quantification was done using the Broad Range RNA kit on a Qubit2.0-Fluorometer (Invitrogen). Each sequencing library was prepared from a single abdomen with the TruSeq^®^ stranded-mRNA sample-prep kit (Illumina) starting with 700 ng total RNA for the *Gmm*^*Sod*+^ and *Gmm*^*Sod*–^ groups (5 replicates/group) and 1000 ng for the *Gmm*^*Sod*^***^–^****^/Sod+^, Gmm^*Sod*^****^–^****^/Ecoli^*^+^, and *Gmm*^*Sod*^***^–^***^/saline^ groups (3 replicates/group). In brief, polyA-mRNA fragments underwent two rounds of purification with poly-T-magnetic beads and were primed with random hexamers for first strand cDNA synthesis. Strand-specificity was preserved by incorporation of dUTP. Library quality was validated using the DNA1000 chip kit on a 21000 Bioanalyzer. Library quantification was performed using the quantitative PCR (qPCR) KAPA library quantification kit (KapaBiosystems) on a LightCycler 480 system (Roche). Libraries were normalized to 2 nM before pooling and 2x100bp paired-end sequenced on an Illumina HiSeq1500 at the Center Medical Genetics (University Antwerp, Belgium). Each library was sequenced over different lanes to minimize lane-to-lane-confounding effects. The sequence data has been submitted to NCBI’s Short Read Archive (BioProject Accession Number PRJNA476840).

### RNA-Seq Data Analysis

Data quality was validated with FastQC (v0.11.416) (Andrews, 2010) and sequencing analysis viewer (SAV) from the Illumina software. Raw reads were mapped with STAR (v2.5.2.b) (Dobin et al., 2013) to the *Glossina morsitans morsitans* reference genome (International Glossina Genome Initiative [IGGI], 2014) (*GmorY1* scaffolds, ASM107743v1, and *GmorY1.5* base features) downloaded from https://www. vectorbase.org (Giraldo-Calderón et al., 2015). Default para- meters were used except for alignIntronMax 5000 and sjdbOverhang 99. Unmapped reads were adapter and quality trimmed with Cutadapt (v1.2.1) (Martin, 2011) and remapped to the reference genome with optimized parameters out- FilterMatchNminOverLread 0.4 and outFilterScoreMinOver- Lread0.4. Mapping statistics were obtained with Log.Final. out. Reads were counted with STAR quantMode.Gene- Counts using the gene dataset *GmorY1.5* (12,969 gene models). The Bioconductor-DESeq2-package (v1.20.0) (Love et al., 2014) was used with default parameters for differential expression analysis. Statistically significant differences were accepted at *p* < 0.05 and adjusted *p*-value for multiple-testing (Benjamini-Hochberg); false discovery rate (FDR) < 10%. Functional annotation of the DEGs was retrieved from VectorBase (*GmorY1.5*, 12,969 predicted transcripts from which 8001 annotated as hypothetical proteins). The GO terms and a second annotation layer were obtained by Blast2GO (Conesa et al., 2005; Götz et al., 2008) using the blastx algorithm to search against the *Drosophila* database (significance cut-off of 1 × 10^–05^). Protein domains and families were identified by querying the Interpro database, integrated in Blast2GO and linked with the GO database. To assess which GO terms were overexpressed relative to the entire transcriptome an enrichment analysis was performed in Blast2GO (Fisher’s exact test; FDR < 0.05). The putative members of *Glossina* innate immune pathways analyzed in this study were obtained as described by Matetovici et al. (2016).

### Total RNA Isolation and qRT-PCR Analysis (cDNA-Based)

Transcriptome validation of the RNA-seq data was done by qRT-PCR using 10 genes identified as differentially expressed between *Gmm*^*Sod*^***^–^****^/Ecoli^*^+^ and *Gmm*^*Sod*^***^–^***^/saline^ in the RNA-seq analysis (primer sequences in Supplementary Additional File 3). For this, the total RNA isolated to prepare the RNA-seq libraries was used (see above). For other experiments, total RNA was isolated with the TRIzol^®^ reagent (Invitrogen) from homogenized abdomens 48 h after bacterial exposure, by performing two rounds of chloroform-phase separation, precipitated with isopropanol, and washed twice with 75%-ethanol. Samples were treated with TurboDNase (Ambion). RNA quantification was done by NanoDrop spectrophotometer and ratios 260/280nm and 260/230nm were determined for RNA purity assessment.

Total RNA was reverse transcribed using Transcriptor ReverseTranscriptase (Roche) and oligo (dT)_15_-primers (Promega). Q-RTPCR amplifications were obtained in duplicate using the SensiMix SYBR No-ROX kit (BioLine) in a total volume of 20 μl and 0.5 μM of each primer (except 0.7 μM for *iap2*) on a LightCycler 480 system (Roche) with following cycling conditions: 10 min./95°C, 40 cycles 10 sec./95°C, 10 sec./ 60°C, and 30 sec./75°C. To select suitable reference genes, the expression of 10 candidate genes was evaluated for stability using total RNA isolated from 10 abdomens per group (*Gmm*^*Sod*^***^–^****^/Sod+^, Gmm^*Sod*^****^–^****^/Ecoli+^, Gmm^*Sod*^****^–^***^/saline^) and performing a geNorm analysis with the qBase+1.5 software (BioGazelle).

### Fluorescence Microscopy

GFP-expressing *S. glossinidius* (10^6^ CFU) or *E. coli* (10^5^ CFU) bacteria were obtained as previously described by De Vooght et al. (2018) and were introduced in 8-day old flies by intrathoracic microinjection and *per os* (see above). At day 2 and 5 after exposure, hemolymph and midguts (i.e., endodermally derived gut tissue flanked by the proventriculus and Malpighian tubules (Dow, 1986)) were collected on a glass slide and analyzed by fluorescence microscopy (Zeiss LSM700).

### Imd-Pathway Activation

On day 8 post-eclosion, flies were exposed to 10^5^ CFU *E. coli* as described above. After 48 h, DNA was isolated (see below) from abdomens to estimate bacterial densities and total RNA was isolated for qRT-PCR expression analysis (see conditions above).

### RNAi-Mediated Imd-Pathway Suppression

To construct the relish dsRNA, the complete *relish* coding sequence (2,599 bp) was first PCR amplified using the Phusion High-Fidelity PCR master mix (New England BioLabs) in a total volume of 50 μl and 0.5 μM of each primer (Supplementary Additional File 4) with following cycling conditions: 30 sec./98°C, 35 cycles 10 sec./98°C, 30 sec./65°C, 30 sec./72°C, and 10 min/72°C. For this, cDNA was generated from total RNA isolated from 10-day old wild-type abdomens as described above. The PCR product was cloned into a ZeroBlunt^TM^ TOPO-plasmid (Invitrogen) and transformed into OneShot^®^TOP10 chemically competent *E. coli* (Invitrogen). The insert sequence was validated by gel electrophoresis and Sanger sequencing. The dsRelish constructs (479 bp) were prepared by *in vitro* transcription (IT) using the Megascript RNAi kit (Ambion). For this, the IT-templates were generated using the Phusion High-Fidelity PCR master mix (New England BioLabs) in a total volume of 50 μl and 0.5 μM of each primer with the primers (Supplementary Additional File 4) with following cycling conditions: 30 s/98°C, 35 cycles 10 s/98°C, 30 s/72°C, 30 s/72°C, and 10 min/72°C. On day 6 post-eclosion, flies (cold-shock anesthetized) were microinjected intrathoracically (34′ gauge Hamilton syringe) with 10 μg dsRelish dissolved in 2–3 μl sterile saline. On day 7 post injection, DNA was extracted (see below) from 10 independent replicates per group to estimate the bacterial densities and total RNA was isolated from fly abdomens for qRT-PCR expression analysis as described above.

### DNA Isolation and *in vivo* Measurement of the Bacterial Densities by qPCR (DNA-Based)

DNA was isolated from abdomens with the EZNA Tissue DNA kit (Omega) and used to estimate bacterial densities with primers for species-specific and single-copy genes (Supplementary Additional File 5). The *S. glossinidius* density was obtained by amplifying a *S. glossinidius*-specific exochitinase-locus as described by De Vooght et al. (2014) using a 1:10 serially diluted standard curve of DNA extracts from a *S. glossinidius* culture (10^2^ CFU/ml – 10^7^ CFU/ml). Because *Wigglesworthia glossinidia* and *Wolbachia* sp. cannot be cultivated, no bacterial standard curves were available for these symbionts and densities were defined as the bacterial genome copy number (resp. *thiamine biosynthesis* and *16S rRNA*) divided by the tsetse host genome copy number (α-*tubulin*). All gene-targets were qPCR (DNA-based) amplified in duplicate as described above using 0.5 μM of each primer, except 0.3 μM for *exochitinase*.

### Trypanosome Infection of Tsetse Flies

Freshly emerged flies were given 24 h post-eclosion a blood meal containing tsetse fly transmissible trypanosome parasites, the pleiomorphic *T. brucei brucei* AnTAR1 strain (Le Ray et al., 1977) or the *T. congolense* MSOROM7 strain (Tihon et al., 2017). For this, bloodstream form parasites were harvested with heparin from cyclophosphamide immune-suppressed mice (Endoxan^®^, Baxter) 6 days post-infection and mixed with defibrinated horse blood to obtain >10^6^ trypanosomes/ml (containing ∼80% intermediate/stumpy-forms for the *T. brucei* infection). Only fully engorged flies were maintained and fed 3 days/week with uninfected blood until dissection. After 28 days, individual flies were analyzed for the presence of procyclic and metacyclic trypanosomes by microscopical examination of their midguts and salivary glands (*T. brucei*) or proboscis (*T. congolense*), respectively. Differences in infection rates between *Gmm*^*Sod*+^ and *Gmm*^*Sod*^***^–^*** flies were compared using Chi-square (two-sided) and considered significant if *p*-values were lower than 0.05. Estimation of the trypanosome abundance in midgut-infected flies was obtained by qPCR analysis using the *18S rRNA-*targeting (105 bp) primers (Supplementary Additional File 6) at 0.5 μM in 20 μl reaction volume and following cycle conditions: 30 s/98°C, 35 cycles 10 s/98°C, 30 s/65°C, 30 s/72°C, and 10 min/72°C.

### Graphs and Statistical Analysis

Graphs were prepared in GraphPadPrism v5.0 and the data is represented as the mean +/− standard deviation. The same software was used for statistical analysis (two-tailed nonparametric *t*-testing for bacterial densities and expression values, Chi-square-testing for the trypanosome infection rate) and *p*-values <0.05 were considered statistically significant.

## Results

### Selective Elimination of *Sodalis glossinidius* From Tsetse Flies

**FIGURE 1:**
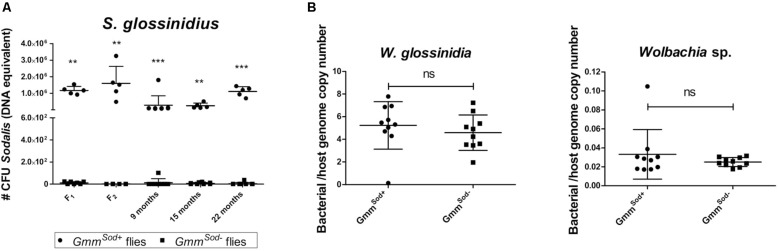
Tsetse fly’s bacterial symbiont densities in offspring flies collected from tsetse females treated with 0.5 μg/mL streptozotocin. **(A)** Building-up the *Gmm*^*Sod–*^ tsetse fly colony using *Sodalis glossinidius*-free flies; flies were further maintained on a normal feeding regime. The *S. glossinidius* density was determined using a standard curve-based *S. glossinidius*-specific qPCR assay on DNA isolated from 10-day old male abdomens (F_1_ and F_2_) and monitored at a 2-week interval in teneral male abdomen of the *Gmm*^*Sod–*^ colony (illustrated by 3 representative timepoints; indicated as months after the females’ F_0_ treatment). *N* = 10 *Gmm*^*Sod–*^ independent replicates and *N* = 5 *Gmm*^*Sod+*^ independent replicates per time point. **(B)** The *Wigglesworthia glossinidia* and *Wolbachia* sp. densities were defined as the bacterial genome copy number over the tsetse host genome copy number (*α-tubulin*) and obtained using species-specific qPCR assays. *N* = 10 independent replicates per group. Values show the bacterial density in each abdomen and are represented as the mean with standard deviation. ^∗∗^*p*-value < 0.01; ^∗∗∗^*p*-value < 0.001; ns: not significant.

The antibiotic streptozotocin, a bacteriocidal analog of N-acetyl glucosamine, the main carbon source utilized by *S. glossinidius* in the fly, has been used to specifically target *S. glossinidius* without affecting *W. glossinidia* (Dale and Welburn, 2001). However, to date the establishment of a *S. glossinidius*-free tsetse fly colony has not been described. In this study, we evaluated the outcome of different streptozotocin treatments, i.e., intrathoracic microinjection, *per os*, or a combination treatment, on the *S. glossinidius* population, tsetse viability and fecundity, and on the *W. glossinidia* and *Wolbachia* sp. populations (summarized in Supplementary Additional Files 1, 2). Supplementation of the bloodmeal with streptozotocin proved to be most effective for clearing *S. glossinidius*, resulting in complete elimination. Intrathoracic microinjection of streptozotocin only resulted in 55% reduction of the *S. glossinidius* population, while combining microinjection and a *per os* treatment also resulted in complete *S. glossinidius* elimination. High doses of streptozotocin (2.5–20 μg/ml) had a detrimental effect on tsetse fly fecundity, with females suffering from damaged reproductive tissues (Supplementary Additional File 7) and an arrest of larval deposition after the first gonotrophic cycle (G_1_). QPCR showed that the antibiotic treatment had no effect on the *W. glossinidia* population, the primary endosymbiont of tsetse known to be important for reproduction. Upon dissection of the sterile females’ reproductive organs we observed atrophy of the ovaries, suggesting that the observed reduction in fecundity resulted from a more direct effect on the host rather than the elimination of its primary symbiont. Finally, continuous supplementation of the bloodmeal with 0.5 μg/ml streptozotocin did not affect female fecundity and resulted in offspring specifically cleared of *S. glossinidius* without affecting tsetse’s other endosymbiotic bacteria *W. glossinidia* and *Wolbachia* sp. (Figure 1B). This allowed us to build up and maintain a tsetse fly colony devoid of *S. glossinidius* only, but with an identical genetic background as the *S. glossinidius* positive colony. The *S. glossinidius*-free status of the colony was monitored throughout the study at a 2-week interval by qPCR and bacterial plating (Figure 1A).

### RNA-Seq Expression Analysis of Innate Immunity-Related Genes in Symbiotic (*Gmm*^*Sod+*^) and *S. glossinidius*-Free (*Gmm*^*Sod–*^) Tsetse Flies

**FIGURE 2:**
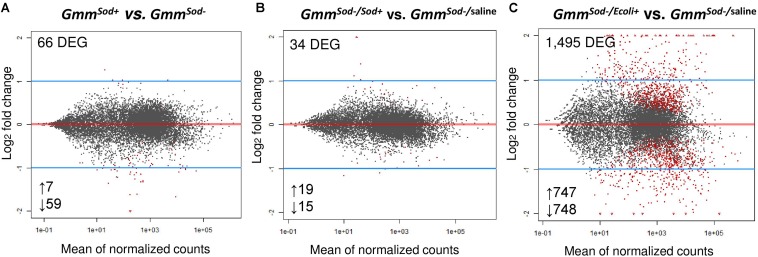
Differentially gene expression patterns obtained by RNA-seq analysis between the tsetse fly groups. **(A)** Comparison of 10-days old symbiotic (*Gmm*^*Sod+*^) versus *S. glossinidius*-free (*Gmm*^*Sod*^*^–^*) flies. **(B)** Comparison of *Gmm*^*Sod*^*^–^* flies exposed to 10^6^ CFU *Sodalis glossinidius* (*Gmm*^*Sod*^*^–^^/Sod^*^+^) versus the corresponding injection-control with sterile saline (*Gmm*^*Sod*^*^–^*^/saline^). **(C)** Comparison of *Gmm*^*Sod*^*^–^* flies exposed to 10^5^ CFU *E. coli* (*Gmm*^*Sod*^*^–^^/Ecoli^*^+^) versus *Gmm*^*Sod*^*^–^^/^*^*saline*^. Bacterial exposure was performed on day 8 post-eclosion via intrathoracic microinjection as well as *per os* and total RNA was extracted after 48 h. Each dot represents the mean of expression (normalized counts) for a given gene. Significantly differentially expressed genes (DEG) were defined by a ***p***-value <0.05 and a false discovery rate (FDR; Benjamini-Hochberg) <10% and are shown in red. Numbers below the plots refer to the amount of up-and downregulated genes.

In an effort to identify genes and pathways that are affected by the presence/absence of the *S. glossinidius* symbiont, we sequenced the whole transcriptome from 10-day old *S. glossinidius*-harboring (*Gmm*^*Sod+*^) and *S. glossinidius*-free (*Gmm*^*Sod*^***^–^***) flies. For this, cDNA libraries were prepared from mRNA isolated from the abdomen of the respective flies and sequenced on an Illumina platform. The sequencing, mapping, and counting results are represented in Supplementary Additional File 8. In total, sequencing yielded 108–202 million high quality reads for the *Gmm*^*Sod+*^ group and 81–148 million for the *Gmm*^*Sod*^***^–^*** group. For each group, a high number of uniquely mapped reads (UMR) was obtained after mapping the raw reads to the *Glossina morsitans morsitans* reference genome (*GmorY1*). This ranged between 85 and 177 million for the *Gmm*^*Sod+*^ group (76–91% of the total raw reads) and 68–125 million for the *Gmm*^*Sod*^***^–^*** group (83–87% of the total raw reads). Differential expression analysis between the *Gmm*^*Sod+*^ and *Gmm*^*Sod*^***^–^*** groups revealed only 66 DEGs out of 10,473 expressed tsetse genes of which the majority (59 genes, 89% of total DEG) was expressed at higher levels in *Gmm*^*Sod*^***^–^*** flies (Figure 2A). None of the identified 66 DEG were associated with putative tsetse innate immune processes. To reveal other processes affected by *S. glossinidius* presence, the DEG sequences were annotated and Gene Ontology (GO) terms were added using Blast2GO (Supplementary Additional File 9). Furthermore, an enrichment analysis was performed but no GO category was identified as enriched in this data set. However, for the 59 downregulated genes, the molecular function category term “structural molecule activity” was vastly present. Of note was the decrease in abundance of five transcripts encoding for chitin-binding proteins, three transcripts encoding for cuticle proteins, and four transcripts involved in chitin synthases and development in *Gmm*^*Sod+*^ flies compared to *Gmm*^*Sod*^***^–^*** flies (Supplementary Additional File 9). Chitin associates with different types of proteins (e.g., structural, enzymes and antibacterial) by non-covalent binding of one or more chitin-binding domains (CBDs) present in their protein sequences (Shen and Jacobs-lorena, 1999). In insects, two main types of CBPs have been identified: the Chitin_bind_4 (pfam 00379) and CBM14 (pfam 00379), also known as the peritrophin-A domain, which is particularly found in the PM proteins of insects and animal chitinases (Elvin et al., 1996; Tetreau et al., 2015). From the five DEG chitin-binding proteins, three contain the Chitin_bind_4 domain and two a peritrophin-A domain (Supplementary Additional File 9).

### RNA-Seq Expression Analysis of Innate Immunity-Related Genes in *Gmm*^*Sod–*^ Flies Exposed to *S. glossinidius* or *E. coli*

Next, we examined whether *S. glossinidius* elicits an immune response after experimental re-exposure in *Gmm*^*Sod–*^ flies (via a combination of injection and *per os*) and compared this to the immune response elicited by a strong immune activator, i.e., *E. coli* (positive control) and the response to pricking stress, i.e., sterile saline injection (negative control). For this, cDNA libraries were prepared from the abdomen of *Gmm*^*Sod–*^ flies 48 h after exposure to 10^6^ CFU *S. glossinidius* (*Gmm*^*Sod–/Sod+*^), 10^5^ CFU *E. coli* (*Gmm*^*Sod–/Ecoli+*^), and sterile saline (*Gmm*^*Sod–/saline*^) and analyzed by RNA-seq. The sequencing, mapping, and counting results are summarized in Supplementary Additional File 10. A total of 805 million high quality reads was obtained across all 9 samples; between 82 and 112 million for the *Gmm*^*Sod–/Sod+*^ group, 82–112 million for the *Gmm*^*Sod*^***^–^****^/Ecoli+^* group, and 77–83 million for the *Gmm*^*Sod–*/saline^ group. A minimum of 87% UMR was obtained after mapping to the *GmorY1* reference genome: 74–97 million for the *Gmm*^*Sod*^***^–^****^/Sod+^* group (87–90% of the total raw reads), 76–99 million for the *Gmm*^*Sod–/Ecoli+*^ group (88–90% of the total raw reads), and 69–74 million for the *Gmm*^*Sod–/saline*^ group (88–90% of the total raw reads). Exposure of *Gmm*^*Sod*^***^–^*** flies to *S. glossinidius* did not evoke broad range transcriptomic alterations in the fly: comparison between the *Gmm*^*Sod*^***^–^****^/Sod+^* and *Gmm*^*Sod–/saline*^ groups revealed only 34 DEG out of 10,476 expressed tsetse genes (Figure 2B) of which none were associated with putative tsetse innate immune processes. Otherwise, exposure of *Gmm*^*Sod*^***^–^*** flies to *E. coli* resulted in 1,495 DEG out of 10,472 expressed tsetse genes compared to the injection-control of which 747 were upregulated (with 29% > 2-fold increase) and 748 downregulated (with 27% < 2-fold decrease) (Figure 2C).

**FIGURE 3:**
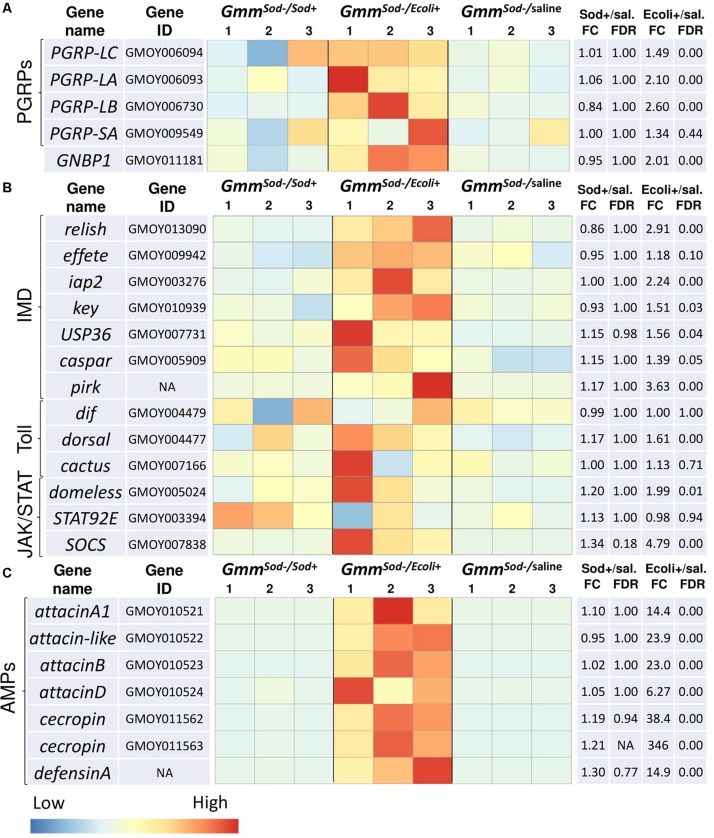
Heat maps showing the transcriptional profiles of innate immunity-related genes obtained by RNA-seq analysis of *S. glossinidius*-free (*Gmm*^*Sod–*^) flies exposed to 10^6^ CFU *Sodalis glossinidius* (*Gmm*^*Sod–/Sod*+^) or 10^5^ CFU *E. coli* (*Gmm*^*Sod–/Ecoli*+^) and their corresponding injection-control with sterile saline (*Gmm*^*Sod–/saline*^). Bacterial exposure was performed on day 8 post-eclosion via intrathoracic microinjection as well as *per os* and total RNA was extracted after 48 h. Genes encoding **(A)** immune-related recognition proteins, **(B)** components of the three major immune signaling pathways; the immune deficiency (Imd)-, Toll-, and JAK/STAT-pathway, and **(C)** antimicrobial peptides (AMPs). Heat maps were obtained by plotting the normalized read counts scaled by row. Colors indicate the *z*-scores ranging from -1 (blue: low expression) to 1 (red: high expression). The biological replicates are indicated as numbers above the columns. Significantly differentially expressed genes (DEG) were defined by a *p*-value <0.05 and a false discovery rate (FDR; Benjamini-Hochberg adjusted *p*-value) <10%. Dif, dorsal-related immunity factor; Ecoli+, *Gmm*^*Sod–/Ecoli*+^; FC, fold change; GNBP1, gram-negative binding protein 1; iap2, inhibitor of apoptosis 2; key, kenny; NA, not applicable; PGRP, peptidoglycan recognition protein; sal., sterile saline-injected flies; SOCS, suppressor of cytokine signaling; Sod+, *Gmm*^*Sod–/Sod*+^; STAT92E, signal transducer and activator of transcription 92E; USP36, ubiquitin-proteasome related protein 36.

In-depth analysis of genes putatively involved in the humoral immune response of the insect (Lemaitre and Hoffmann, 2007; Weiss et al., 2008) was performed by examining the expression of pattern recognition encoding genes, genes encoding components of the major insect immune signaling pathways (Imd-, Toll-, and JAK/STAT-pathway), and immune effector encoding genes (AMPs). Three of the four analyzed genes encoding PGRPs were upregulated upon *E. coli* exposure: an increase of 1.5-fold for *PGRP-LC* (GMOY006094) and 2.1-fold for *PGRP-LA* (GMOY006093), i.e., two PGRPs associated with Gram-negative bacterial sensing upstream of the Imd-pathway, and a 2.6-fold increase of *PGRP-LB* (GMOY006730), a negative regulator of the Imd-pathway (Figure 3A). Also, a recognition protein encoding gene belonging to the Gram-negative binding (GNBP) protein family and associated with the Toll-pathway, *GNBP1* (GMOY011181), was 2.0-fold increased in expression in response to *E. coli*, whereas no significant increase was observed of *PGRP-SA* (GMOY009549) which often co-operates with GNBP1 (Figure 3A). In contrast to *E. coli*, flies exposed to *S. glossinidius* showed no significant up-or downregulation of immune recognition protein encoding genes at 48 h after challenge in the RNA-seq analysis (Figure 3A).

Many genes encoding key components of the Imd-pathway were significantly increased in expression in response to *E. coli* but not in response to *S. glossinidius*, including the transcription factor *relish* (GMOY013090), the inhibitor of κB kinase (IKK) complex constituent *kenny* (*key; GMOY010939*), *effete* (GMOY009942), and the ubiquitination machinery components inhibitor of apoptosis 2 (*iap2;* GMOY003276) (Figure 3B). Multiple Imd-pathway regulatory protein encoding genes were also increased upon *E. coli* exposure, including a *ubiquitin-specific protease* (*USP36*; GMOY007731), *caspar* (GMOY005909), and *pirk* (not annotated in the *GmorY1* assembly) (Figure 3B). Except *dorsal* (GMOY004477), no genes encoding proteins of the Toll-pathway were significantly up-or downregulated in our study following *E. coli* or *S. glossinidius* exposure (Figure 3B), indicating that this pathway is not significantly induced by Gram-negative bacteria in the fly. Components of the JAK/STAT-pathway that showed increased expression in response to *E. coli* include a cytokine receptor encoding gene (GMOY005024; a possible *domeless* ortholog) and the negative regulator *SOCS* (GMOY007838), whereas expression of the JAK/STAT-pathway’s regulatory protein encoding gene *STAT92E* (GMOY003394) was not affected by *E. coli* exposure and no JAK/STAT-pathway components were affected by *S. glossinidius* exposure in the fly (Figure 3B).

We found that downstream immune effector genes encoding different AMPs such as *attacin* (four different genes: GMOY010521, GMOY010522, GMOY010523, and GMOY010524), *cecropin* (two different genes: GMOY011562 and GMOY011563), and *defensinA* (not annotated in the *GmorY1* assembly) were highly increased in expression upon exposure to *E. coli* while none were affected by the *S. glossinidius* symbiont (Figure 3C).

The outcome of the above described RNA-seq analysis was validated with qRT-PCR by analyzing the relative expression of a subset of 10 tsetse fly genes that were identified as up-or downregulated, or unaffected, in the RNA-seq analysis (primers in Supplementary Additional File 3). The same RNA extracted for the RNA-seq library construction was used for this analysis. Comparison of the fold changes in expression showed a high similarity between the two methods, resulting in a Pearson’s value of 0.99 which confirms the validity of our RNA-seq analysis (Supplementary Additional File 11).

### Targeted qRT-PCR Expression Analysis of Innate Immunity-Related Genes in *Gmm*^*Sod–*^ Flies Exposed to *S. glossinidius* or *E. coli*

**FIGURE 4:**
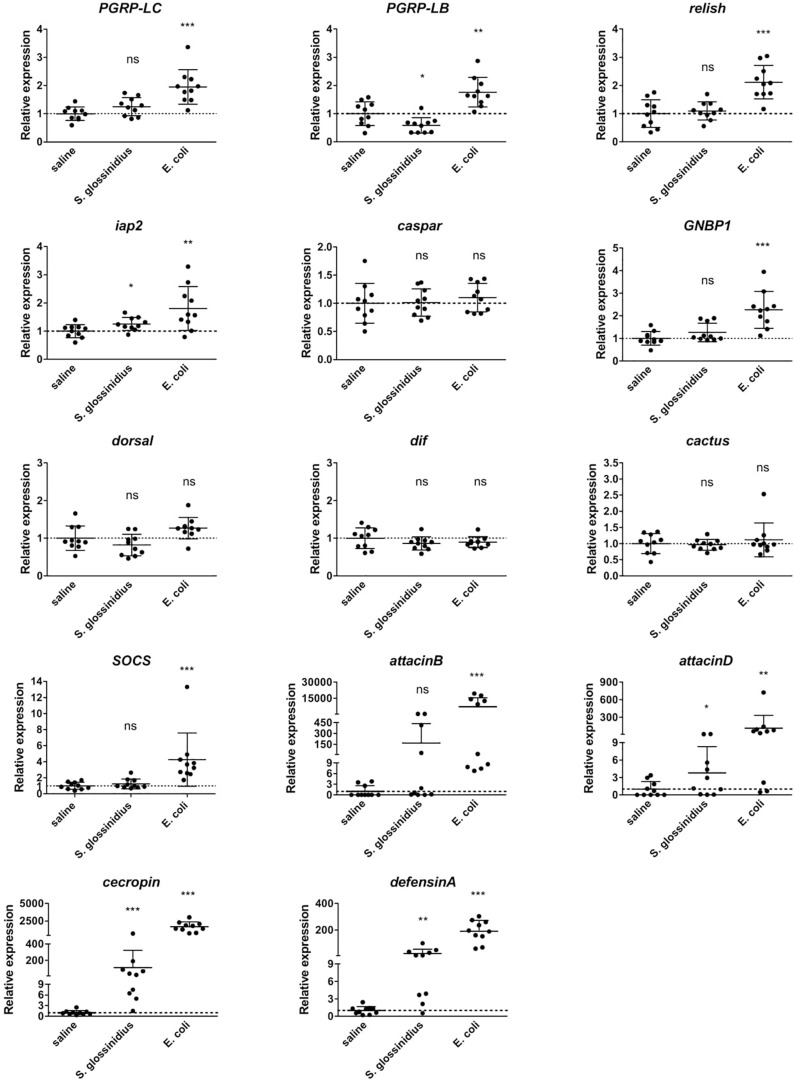
Relative expression levels of innate immunity-related genes obtained by qRT-PCR analysis in *S. glossinidius*-free (*Gmm*^*Sod–*^) flies exposed to 10^6^ CFU *Sodalis glossinidius* or 10^5^ CFU *E. coli* and their corresponding injection-control with sterile saline. Bacterial exposure was performed on day 8 post-eclosion via intrathoracic microinjection as well as *per os* and total RNA was extracted after 48 h. The expression levels were normalized against the two tsetse reference genes *β-tubulin* (GMOY000148) and *pleiotrophic regulator 1* (GMOY006161). Expression in each abdomen is plotted relative to the mean of the injection-control group. Values are represented as the mean with the standard deviation. *N* = 10 independent replicates per group. ^*^p-value < 0.05, ^∗∗^*p*-value < 0.01, ^∗∗∗^*p*-value < 0.001; Dif, dorsal-related immunity factor; GNBP1, gram-negative binding protein 1; iap2, inhibitor of apoptosis 2; ns, not significant; PGRP, peptidoglycan recognition protein; SOCS, suppressor of cytokine signaling.

The transcriptional profile of innate immunity-related genes in *Gmm*^*Sod*^***^–^*** flies following exposure to *S. glossinidius* or *E. coli* was further investigated with a targeted qRT-PCR expression analysis using cDNA isolated from 10 individual abdomens per group (primers in Supplementary Additional File 3). Similar as observed in the RNA-seq analysis, the expression levels of the pattern recognition encoding genes *PGRP-LC* and *PGRP-LB* were significantly increased in response to *E. coli* compared to the response to sterile saline (Figure 4). *S. glossinidius* exposure on the other hand, did not evoke different expression levels of the Imd receptor *PGRP-LC* but resulted in a moderate decrease of the negative regulator *PGRP-LB* (1.7-fold) (Figure 4). The Imd-pathway components *relish* and *iap2* were significantly increased in flies upon *E. coli* exposure and a moderate increase in expression of *iap2* (1.3-fold) was detected in response to *S. glossinidius* (Figure 4). We observed a 2.3-fold increase in expression of the recognition receptor acting upstream of Toll, i.e., *GNBP1*, in response to *E. coli*, whereas the other Toll-pathway components showed no altered expression levels and no Toll-pathway components were affected by *S. glossinidius* exposure (Figure 4). The JAK/STAT-pathway regulator *SOCS* was 4.3-fold increased in response to *E. coli* but not to *S. glossinidius* (Figure 4). The expression of AMP-encoding genes *attacinB*, *attacinD*, *cecropin*, and *defensinA* were all highly increased upon *E. coli* exposure and at lower but significant levels in response to *S. glossinidius*, with a high variability observed between the replicates (Figure 4).

### Expression of Innate Immunity-Related Genes in *Gmm*^*Sod*–^ Flies Exposed to Different *S. glossinidius* Concentrations

**FIGURE 5:**
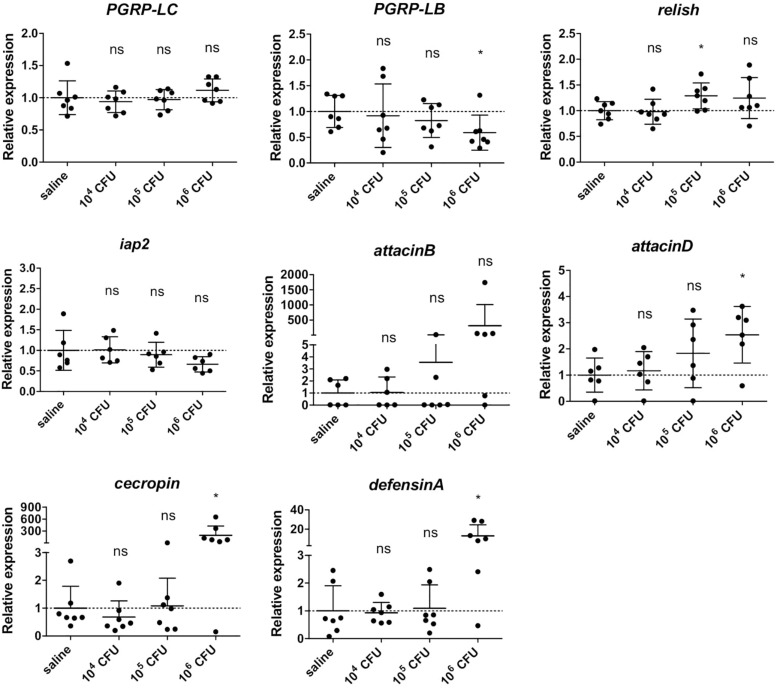
Relative expression levels of innate immunity-related genes in the abdomen of *S. glossinidius*-free (*Gmm*^*Sod–*^) flies exposed to 10^4^ CFU, 10^5^ CFU, or 10^6^ CFU *Sodalis glossinidius* and their corresponding injection-control with sterile saline. Bacterial exposure was performed on day 8 post-eclosion via intrathoracic microinjection as well as *per os* and total RNA was extracted after 48 h. The expression levels were obtained by qRT-PCR analysis and normalized against the two tsetse reference genes *β-tubulin* (GMOY000148) and *pleiotrophic regulator 1* (GMOY006161). Expression in each abdomen is plotted relative to the mean of the injection-control group. Values are represented as the mean with the standard deviation. *N* = 7 independent replicates per group. ^*^p-value < 0.05; iap2, inhibitor of apoptosis 2; ns, not significant; PGRP, peptidoglycan recognition protein.

We then determined whether the above described immune response in *Gmm*^*Sod*^***^–^*** flies to *S. glossinidius* was dependent on the exposed *S. glossinidius* concentration. As we know from previous studies that exposing flies to 10^7^ CFU *S. glossinidius* results in a high mortality (De Vooght et al., 2014), flies were exposed to 10^4^ CFU, 10^5^ CFU, or 10^6^ CFU *S. glossinidius* and the transcriptional profile of immunity-related genes was determined by qRT-PCR 48 h post challenge (primers in Supplementary Additional File 3). We found that the expression of *PGRP-LC* was not affected in flies exposed to any of the *S. glossinidius* concentrations compared to the control flies injected with sterile saline (Figure 5), confirming our previous findings that the *S. glossinidius* symbiont does not upregulate the Imd-pathway receptor. Only when flies were exposed to the highest dose of 10^6^ CFU *S. glossinidius*, a downregulation of the Imd-pathway regulator *PGRP-LB* was observed (Figure 5). In terms of effector molecules, expression of the AMP-encoding genes *cecropin* and *defensinA* was only increased in flies that were exposed to 10^6^ CFU *S. glossinidius*, while exposing flies to lower doses, i.e., 10^4^ CFU and 10^5^ CFU, did not affect AMP expression (Figure 5). These findings demonstrated a threshold-dependent immune response of the tsetse fly to the *S. glossinidius* symbiont, with only exposure to the high dose of 10^6^ CFU resulting in a moderate immune response in the fly.

### Expression of Innate Immunity-Related Genes in *Gmm*^*Sod–*^ Flies Exposed to a Non-tsetse Derived *Sodalis* Strain

**FIGURE 6:**
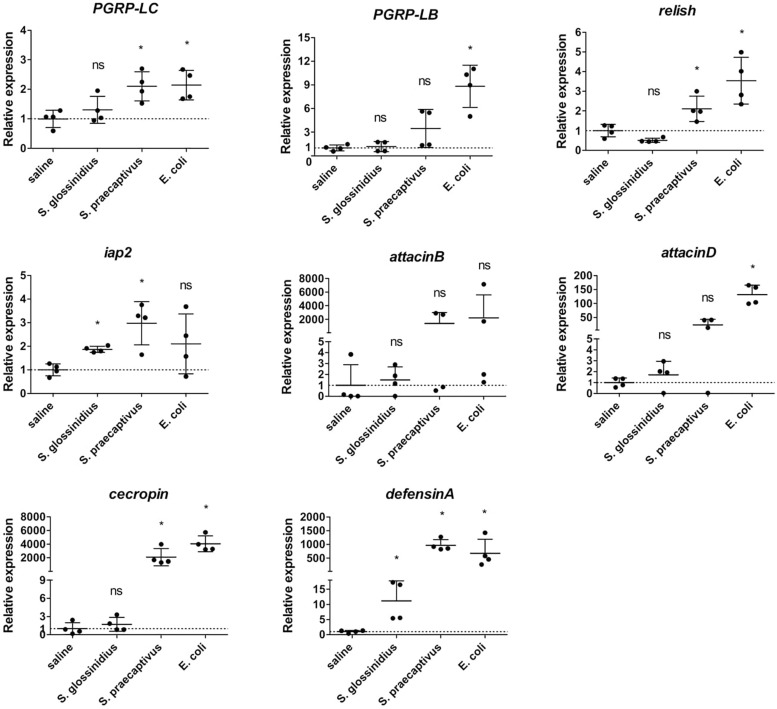
Relative expression levels of innate immunity-related genes in the abdomen of *S. glossinidius*-free (*Gmm*^*Sod–*^) flies exposed to 10^6^ CFU *Sodalis glossinidius*, 10^6^ CFU *Sodalis praecaptivus*, or 10^5^ CFU *E. coli* and their corresponding injection-control with sterile saline. Bacterial exposure was performed on day 8 post-eclosion via intrathoracic microinjection as well as *per os* and total RNA was extracted after 48 h. The expression levels were obtained by qRT-PCR analysis and normalized against the two tsetse reference genes *β-tubulin* (GMOY000148) and *pleiotrophic regulator 1* (GMOY006161). Expression in each abdomen is plotted relative to the mean of the injection-control group. Values are represented as the mean with the standard deviation. *N* = 4 independent replicates per group. ^*^p-value < 0.05; iap2, inhibitor of apoptosis 2; ns, not significant; PGRP, peptidoglycan recognition protein.

The availability of *S. praecaptivus*, a close non-insect associated relative of *S. glossinidius*, allowed us to compare host immune responses toward *S. glossinidius* and an environmental precursor of the *Sodalis*-allied clade of insect symbionts. For this, we exposed *Gmm*^*Sod*^***^–^*** to 10^6^ CFU *S. glossinidius* or 10^6^ CFU *S. praecaptivus* and examined the transcriptional profile of immunity-related genes by qRT-PCR 48 h post challenge (primers in Supplementary Additional File 3). In contrast to *S. glossinidius*, flies exposed to *S. praecaptivus* showed a 2.1-fold increase in expression of *PGRP-LC* compared to the expression in control flies, which was very similar as the level obtained in response to *E. coli* (Figure 6). Expression of *PGRP-LB* was not different upon *S. praecaptivus* exposure and *relish* and *iap2* were resp. 2.1-fold and 2.3-fold increased (Figure 6). The AMP-encoding genes *attacinD*, *cecropin*, and *defensinA* were highly increased in expression in response to *S. praecaptivus* (Figure 6). These results demonstrated that, unlike *S. glossinidius*, the non-tsetse derived *S. praecaptivus* strain is effectively recognized by the Imd-pathway and leads to AMP expression at a similar level compared to flies exposed to the strong immune activator *E. coli.*

### The Presence of *S. glossinidius* in the Tsetse Fly Does Not Affect Its Capacity to Mount an Immune Response After Bacterial Exposure

**FIGURE 7:**
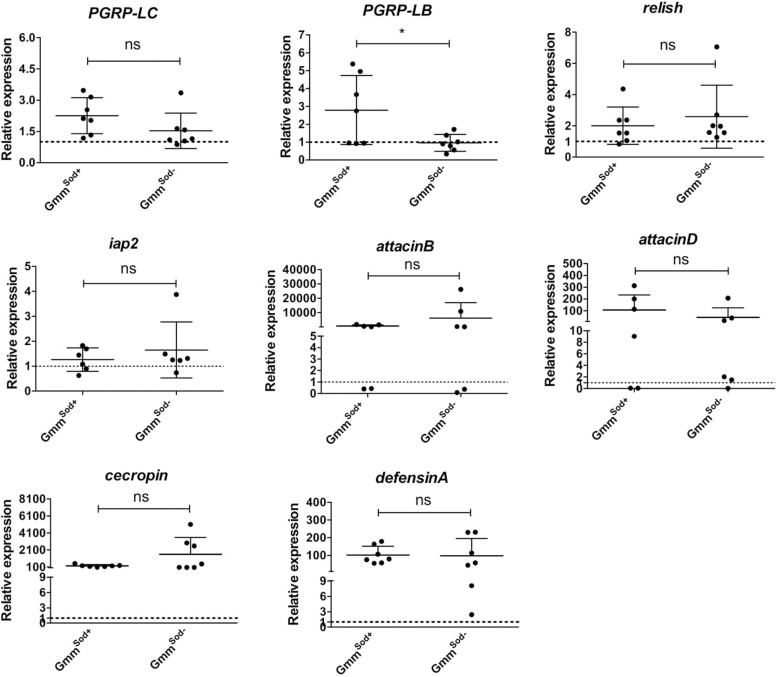
Relative expression levels of innate immunity-related genes in the abdomen of symbiotic (*Gmm*^*Sod*+^) and *S. glossinidius*-free (*Gmm*^*Sod*^*^–^*) flies exposed to 10^5^ CFU *E. coli.* Bacterial exposure was performed on day 8 post-eclosion via intrathoracic microinjection as well as *per os* and total RNA was extracted after 48 h. The expression levels were obtained by qRT-PCR analysis and normalized against the two tsetse reference genes *β-tubulin* (GMOY000148) and *pleiotrophic regulator 1* (GMOY006161). Expression in each abdomen is plotted relative to the mean of the injection-control groups in *Gmm*^*Sod*+^ and *Gmm*^*Sod*^*^–^* flies resp. Values are represented as the mean with the standard deviation. *N* = 7 independent replicates per group. ^*^p-value < 0.05; iap2, inhibitor of apoptosis 2; ns, not significant; PGRP, peptidoglycan recognition protein.

Here we evaluated whether the observed absence of a host immune response to *S. glossinidius* is not due to an active suppression of the immune system by the symbiont. Therefore, we investigated whether the presence of an established *S. glossinidius* population has an impact on the capacity of the tsetse fly to mount an immune response to the strong immune activator *E. coli*. For this, *Gmm*^*Sod+*^ and *Gmm*^*Sod*^***^–^*** flies were exposed to *E. coli* via intrathoracic microinjection and *per os* and the transcriptional profile of immunity-related genes was assessed by qRT-PCR after 48 h (primers in Supplementary Additional File 3). We found that the expression levels of *PGRP-LC, relish, iap2*, and the AMPs *attacinB, attacinD, cecropin*, and *defensinA* after *E. coli* exposure were similar in *Gmm*^*Sod+*^ and *Gmm*^*Sod*^***^–^*** flies (Figure 7), indicating that the presence of *S. glossinidius* in the fly does not affect its capacity to activate the immune signaling pathway or expression of downstream effector encoding genes.

### The *S. glossinidius* Population in the Tsetse Fly Is Not Susceptible to an Imd-Mediated Immune Response

**FIGURE 8:**
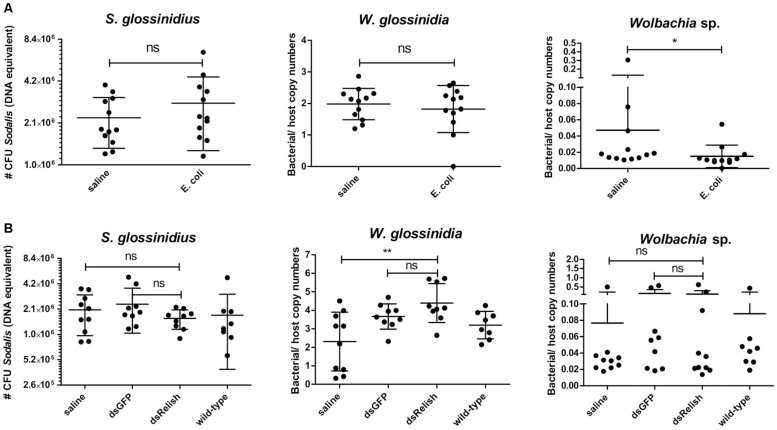
**(A)** *Sodalis glossinidius*, *Wigglesworthia glossinidia*, and *Wolbachia* sp. densities in tsetse flies with an activated immune deficiency (Imd)-pathway, derived by exposing flies on day 8 post-eclosion to 10^5^ CFU *E. coli* via intrathoracic microinjection as well as *per os* and evaluated in the abdomen after 48 h, as well as their corresponding injection-control with sterile saline. **(B)** *S. glossinidius*, *W. glossinidia*, and *Wolbachia* sp. densities in the tsetse flies with an impaired Imd-pathway, derived 7 days after subjecting flies to a dsRelish-treatment, their corresponding controls with dsGFP and sterile saline, and untreated, age-matched wild-type counterparts. The RNA interference (RNAi)-experiment was performed in duplicate and the results are shown from one experiment. The *S. glossinidius* density was determined using a standard curve-based *S. glossinidius*-specific qPCR assay. The *W. glossinidia*and *Wolbachia* sp. densities were defined as the bacterial genome copy number over the tsetse host genome copy number (*α-tubulin*) and obtained using species-specific qPCR assays. Values show the bacterial density in each abdomen and are represented as the mean with the standard deviation. *N* = 10 independent replicates per groups and *N* = 8 independent wild-type flies. The number *S. glossinidius* CFU is represented in log-scale on the y-axis. ^*^p-value < 0.05; ^∗∗^*p*-value < 0.01; ns, not significant.

**FIGURE 9:**
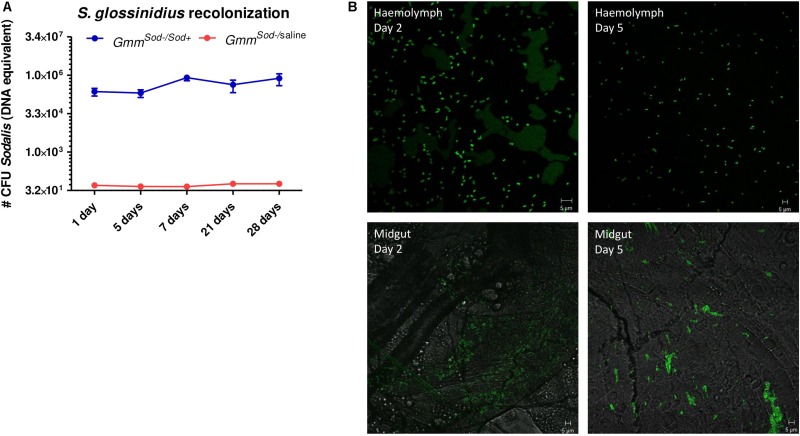
*Sodalis glossinidius* recolonization in the tsetse fly assessed after exposing *S. glossinidius*-free flies to 10^6^ CFU *S. glossinidius* on day 8 post-eclosion via intrathoracic microinjection and *per os* (*Gmm*^*Sod–/Sod*+^). **(A)** Monitoring of the *S. glossinidius* density using a standard curve-based *S. glossinidius*-specific qPCR assay on DNA isolated from the fly abdomen at multiple time points after exposure; *N* = 8 independent replicates per time point. As a control, the *S. glossinidius* density was obtained in flies injected with sterile saline (*Gmm*^*Sod–/saline*^); *N* = 3 independent replicates per time point. Values show the bacterial density in each abdomen and are represented as the mean with the standard deviation. The number *S. glossinidius* CFU is represented in log-scale on the y-axis. **(B)** Visualization of *S. glossinidius* bacteria in the tsetse fly hemolymph and midgut two and five days after exposure.

To evaluate whether the growth of *S. glossinidius* is affected by the host immune system, we monitored the effects of attenuating or activating tsetse immunity on *S. glossinidius* densities in the fly. The Imd-pathway was induced by subjecting flies to *E. coli* via intrathoracic microinjection and *per os*, which was validated by an increased expression of *PGRP-LC*, *PGRP-LB*, *relish*, and the AMPs *cecropin*, *attacinB*, and *defensinA* 48 h after exposure (Supplementary Additional File 12). We then used qPCR to measure the effect of this immune activation on the *S. glossinidius* population in the fly abdomen and showed that the *S. glossinidius* density was not affected by Imd-pathway activation (Figure 8A). This was further evidenced by the fact *S. glossinidius* was able to recolonize *Gmm*^*Sod*^***^–^*** flies, even after the introduction of the immune-activating threshold of 10^6^ CFU bacteria. Indeed, during the 28 days observation period, the *S. glossinidius* density remained at high levels (approximately 8.0 × 10^5^ CFU) after introducing 10^6^ CFU *S. glossinidius* via intrathoracic microinjection and *per os* (Figure 9A). This clearly demonstrated that the introduced *S. glossinidius* bacteria remained viable and were able to establish a stable population in the fly. This was also illustrated by the reintroduction of GFP-tagged *S. glossinidius* bacteria that were widely abundant in the fly hemolymph and midgut at different time points after the reintroduction (e.g., day 5; Figure 9B) while GFP-tagged *E. coli* were nearly undetectable (data not shown). We did not examine the presence of *S. glossinidius* in other tissues of *Gmm*^*Sod*^***^–^*** challenged flies.

Next, we determined whether the *S. glossinidius* population was affected in flies with an impaired immune function. For this we used RNAi to knockdown the expression of *relish*, the transcriptional activator at the downstream end of the Imd-pathway. Using this methodology, we were able to repress transcription of *relish* with 48% resulting in a reduced expression of multiple AMPs: 99% for *attacinB*, 98% for *cecropin*, and 96% for *defensinA* on day 7 after dsRelish treatment (Supplementary Additional File 13). Measurement of the *S. glossinidius* density with qPCR showed no alteration in the bacterial density between immune impaired flies and flies exposed to the injection-control of sterile saline (Figure 8B); this was confirmed in a second, independent RNAi-experiment. Collectively, these results show that *S. glossinidius* is not susceptible to the tsetse fly’s immune responses indicating that the humoral immune pathway does not play a major role in controlling *S. glossinidius* proliferation in the fly.

### Impact of *S. glossinidius* Presence on *Trypanosoma* sp. Infection Rate and Density in the Tsetse Fly

**FIGURE 10:**
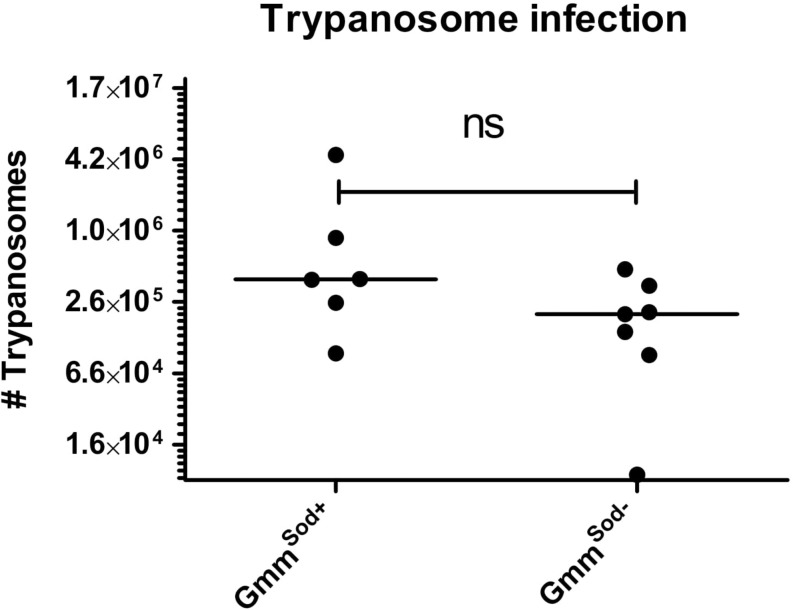
Trypanosome infection density in the tsetse fly midgut of symbiotic (*Gmm*^*Sod+*^) and *S. glossinidius*-free (*Gmm*^*Sod–*^) flies 28 days after they received a *Trypanosoma brucei brucei*-parasitized blood meal one day post-eclosion. The trypanosome density was determined using a standard curve-based trypanosome-specific qPCR assay. Values show the density in each midgut and are represented as the median per group. *N* = 6 independent replicates for the *Gmm*^*Sod+*^ group and *N* = 7 independent replicates for the *Gmm*^*Sod–*^ group. The number of trypanosomes is represented in log-scale on the y-axis. Ns, not significant.

Finally, we investigated whether the *S. glossinidius* presence/absence has an impact on the tsetse fly’s susceptibility to trypanosomes. Therefore, freshly emerged *Gmm*^*Sod+*^ and *Gmm*^*Sod*^***^–^*** flies were fed a first blood meal supplemented with *Trypanosoma brucei brucei* blood stream form trypanosomes and the infection outcome was determined at the level of the midgut and salivary glands. In a first experiment, resp. 39.7 and 32.4% of *Gmm*^*Sod+*^ flies and *Gmm*^*Sod*^***^–^*** flies showed a midgut trypanosome infection (*p* = 0.18) (Table 1, Experiment 1). In two following independent infection experiments, flies were also verified for the maturation rate into a salivary gland infection. No difference in the maturation of procyclic trypomastigotes into the infectious metacyclic stage in the tsetse fly salivary glands was observed between *Gmm*^*Sod+*^ and *Gmm*^*Sod*^***^–^*** flies (Table 1, Experiments 2–3). Furthermore, the presence of *S. glossinidius* had no significant impact on the amount of trypanosome parasites established in the tsetse fly midgut (*p* = 0.10) (Figure 10). Additionally, freshly emerged *Gmm*^*Sod+*^ and *Gmm*^*Sod*^***^–^*** flies were fed a first blood meal supplemented with *T. congolense*. Also here, no difference in infection outcome was observed between *Gmm*^*Sod+*^ and *Gmm*^*Sod*^***^–^*** flies at the midgut (*p* = 0.46) or mouthpart (proboscis) level (*p* = 0.84) (Table 2). Together, the results from these experiments showed that the establishment of a trypanosome infection in the tsetse fly midgut and their subsequent maturation in the salivary glands were not affected by the presence of *S. glossinidius*.

**TABLE 1:** Trypanosome infection outcome in tsetse fly midguts (MG) and salivary glands (SG) of symbiotic (*Gmm*^*Sod*+^) and *S. glossinidius*-free (*Gmm*^*Sod*–^) flies that were given a *Trypanosoma brucei brucei*-parasitized blood meal when they were newly emerged, 1 day post-eclosion.

	**Symbiont status fly**	**# MG infected/total flies**	**Infection rate MG (%)**	**Chi-sq/*p*-value**	**# SG infected/MG-infections**	**Infection rate SG (%)**	**Chi-sq/*p*-value**
Experiment 1	*Gmm*^*Sod+*^	29/73	39.7	0.18	NA	NA	NA
	*Gmm*^*Sod*^***^–^***	22/68	32.4		NA	NA	
Experiment 2	*Gmm*^*Sod+*^	6/71	8.5	0.72	4/6	66.7	0.93
	*Gmm*^*Sod*^***^–^***	7/70	10.0		4/7	57.1	
Experiment 3	*Gmm*^*Sod+*^	20/83	24.1	0.12	12/20	60.0	0.31
	*Gmm*^*Sod*^***^–^***	12/83	14.5		5/12	41.7	

*NA, not applicable.*

**TABLE 2:** Trypanosome infection outcome in tsetse fly midguts (MG) and proboscis (prob) of symbiotic (*Gmm*^*Sod*+^) and *S. glossinidius*-free (*Gmm*^*Sod*–^) flies that were given a *Trypanosoma congolense*-parasitized blood meal when they were newly emerged, 1 day post-eclosion.

**Symbiont status fly**	**# MG infected/total flies**	**Infection rate MG (%)**	**Chi-sq/*p*-value**	**# Prob infected/MG-infections**	**Infection rate Prob (%)**	**Chi-sq/*p*-value**
*Gmm*^*Sod+*^	19/119	16.0	0.46	18/19	94.7	0.84
*Gmm*^*Sod–*^	25/128	19.5		24/25	96	

## Discussion

Recent years have seen an increasing interest in understanding which immunological mechanisms allow insects to maintain a balance between preserving a beneficial microbiome and protecting against pathogens. However, when multiple symbionts coexist in the same host, studying the immunological relationship between a specific symbiont and its host can be very challenging. Therefore, establishing experimental insect lines lacking or harboring a specific symbiont represents a powerful tool that enables to investigate rigorously the role of a given symbiont in complex systems where an insect harbors multiple endosymbiotic bacteria (Koga et al., 2007; Zhang et al., 2015). In this study we described the establishment of a *S. glossinidius*-free (*Gmm*^*Sod–*^) tsetse fly line by crossing the *S. glossinidius*-free offspring from pregnant female flies that were treated with a *S. glossinidius*-specific antibiotic streptozotocin without affecting the tsetse’s other bacterial symbionts, i.e., *W. glossinidia* and *Wolbachia* sp. The availability of these *S. glossinidius*-free flies allowed us to investigate in-depth the interaction between *S. glossinidius* and the tsetse fly’s immune system and its impact on the fly’s vector competence for African trypanosomes. Comparative transcriptome analysis of *S. glossinidius*-harboring (*Gmm*^*Sod*+^) versus *S. glossinidius*-free (*Gmm*^*Sod–*^) flies showed a global picture of the genes that were affected in response to the symbiont’s presence. Only a limited number of tsetse genes were differentially expressed as a result of *S. glossinidius* presence of which the majority (89% of total DEG) showed a decreased expression in *Gmm*^*Sod*+^ flies. Although no significantly enriched GO terms were identified, category terms like structural constituent of chitin, chitin-binding, and cuticle chitin biosynthetic process were vastly present. Downregulation of cuticular and chitin-related proteins has also been described in pea aphids infected with *Serratia symbiotica* compared to an uninfected matriline (Burke and Moran, 2011) and in pea aphids infected by *Buchnera* symbionts compared to *Buchnera*-cured aphids (Wang et al., 2010), suggesting a more common response. It is noteworthy here that the opposite was observed in the *Anopheles coluzzii* mosquito where the gut microbiota induces the expression of several components of the peritrophic matrix (PM) required for the synthesis of a structurally complete PM (Rodgers et al., 2017).

It remains unclear what the biological impact is for the tsetse fly of the decrease in expression of structural protein-encoding genes in *S. glossinidius*-harboring flies. Chitin is a linear biopolymer of N-acetyl-glucosamine (GlcNAc) and a crucial component of the insect exoskeleton, the PM in the midgut, and the cuticle lining the foregut and hindgut. Next to this, GlcNAc is also one of the major components of bacterial cell wall peptidoglycan and the principle carbon source utilized by *S. glossinidius* during its growth (Dale and Maudlin, 1999). Chitin associates with different types of proteins such as the peritrophins that are important constituents of the insect PM. In tsetse, the PM plays a important role as an infection barrier for trypanosomes (Aksoy et al., 2016) and the tsetse’s larval microbiota has been shown to contribute to its proper development (Weiss et al., 2013). So far, no functional studies have been carried out on *S. glossinidius* in the context of the fly’s PM but it is worth mentioning that a proteome analysis of the tsetse PM identified the presence of *S. glossinidius* proteins (Rose et al., 2014), suggesting a close association with this bacterium. Here, it could be plausible that the presence of *S. glossinidius* affects the structural integrity of the PM in the fly but more functional research is required to explore this further.

Despite the abundant presence of *S. glossinidius* within the tsetse fly hemolymph and midgut, the transcript abundance of immunity-related genes was not significantly affected. This suggests the existence of an immune tolerance mechanism toward this symbiont which is a prerequisite to establish an effective infection. Indeed, to allow the establishment of a sustained relationship with their host, facultative symbionts should not be perceived as hostile by the host immune system. Here, the ability to cultivate *S. glossinidius* outside its host allowed us to investigate more in-depth how *S. glossinidius* is perceived by the tsetse immune system compared to a pathogenic enterobacterium *E. coli*. For this, we examined the immune responses in *Gmm*^*Sod*–^ flies to infection by cultured *S. glossinidius* and *E. coli* using RNA-seq and qRT-PCR. Analysis of genes involved in immunity demonstrated that *S. glossinidius* did not activate a systemic immune response in *Gmm*^*Sod*–^ flies in contrast to exposure with exogenous *E. coli* that resulted in a full immune activation mediated by peptidoglycan recognition protein PGRP-LC. This observed upregulated AMP expression in response to *E. coli* in our study is consistent with previous studies where injection of *E. coli* resulted in the expression of multiple AMPs including *defensin*, *attacin* and *cecropin* thus confirming the strong immunogenic nature of an *E. coli* infection in tsetse flies (Hao et al., 2001; Boulanger et al., 2002). Only when exceeding a threshold exposure of 10^6^ CFU *S. glossinidius*, *Gmm*^*Sod*–^ flies elicited a moderate immune response. Exposure of *Gmm*^*Sod*–^ flies to 10^5^ CFU heat-inactivated *E. coli* also resulted in a strong immune response (data not shown), suggesting that the higher immune response of the flies to *E. coli* does not result from a higher abundance of MAMPs due to the higher cell division rate of *E. coli*, but rather due to MAMPs structural divergence between *S. glossinidius* and *E. coli*. In addition, we also demonstrated that the absence or presence of *S. glossinidius* in the tsetse fly does not alter the elevated immune effector response following exposure to *E. coli*. Conclusively, these results clearly show that the absence of a tsetse immune response to *S. glossinidius* is not due to a *S. glossinidius*-mediated suppression of the immune system but rather the result of a compromised detection of *S. glossinidius* by the tsetse immune recognition mechanism.

Our results demonstrate substantial and significant individual variation in expression of AMP encoding genes in the bacteria exposed groups but especially after an *E. coli* challenge. Here, the expression levels of the AMPs were found to differ by more than 100- and 1000-fold between individual flies for *attacinD* and *attacinB* respectively (Figure 4), despite controlling for age and time of infection. This finding is not unexpected and high variation in immune gene expression after ectopic infection among individual members from the same colony has also been reported in *D. melanogaster* (Hoekstra et al., 2004), honeybees (Evans and Pettis, 2005), and in *B. terrestris* (Riddell et al., 2009). Our experimental setup does not allow analyzing the underlying causes for the observed variation, but whatever these are they could be of importance in the context of vector competence. Indeed, although tsetse flies are the sole vectors of African trypanosomes, they generally are refractory to infection, with estimates of < 1% of flies having trypanosome-infected salivary glands, even in endemic areas. The innate immune responses, particularly the AMPs regulated via the Imd pathway, are among the factors that have been suggested to contribute to tsetse’s refractoriness to trypanosome transmission (Hu and Aksoy, 2006). Further studies are needed to investigate the underlying mechanisms that drive individual variability in immunocompetence and whether this plays an important role in the refractoriness of the flies to trypanosome infection.

Since host fitness costs are most likely minimized by limiting excessive endosymbiont proliferation, the factors that limit endosymbiont proliferation are of high relevance to understand how host-symbiont coevolution has shaped the host immune response. The observation that *S. glossinidius* elicits a moderate immune response only above a threshold density could indicate that the tsetse immune system plays a role in controlling the *S. glossinidius* densities to maintain it at a steady-state optimal level. Indeed, the proliferation of bacteria in the insect host has been shown to be controlled by the host immune system. For example, the proliferation and localization of the primary symbiont of the *Sitophilus* weevil, *Sodalis pierantonius*, is controlled by a specific antimicrobial peptide, Coleoptericin A (ColA) (Login et al., 2011). ColA was shown to function as a “molecular guard” by preventing the symbiont from escaping the bacteriome. This compartmentalization strategy has recently been shown to be under the control of the same Imd-like pathway that regulates AMP expression upon bacterial infection (Maire et al., 2018). Interestingly, Imd-pathway activation and AMP production in response to ectopic infection did not interfere with the *S. pierantonius* endosymbiont load (Masson et al., 2015), implying that the bacteriome possesses an immune program adapted to maintaining endosymbiotic homeostasis under standard conditions while retaining the ability to mount an immune response against exogenous microbial intruders without affecting its primary symbiont (Zaidman-Rémy et al., 2018). In our study, we monitored the effects of attenuating or activating the tsetse immune system on the *S. glossinidius* densities in the fly. RNAi-mediated immune suppression did not affect the *S. glossinidius* population nor did experimental activation of the Imd-pathway, indicating that *S. glossinidius* is not susceptible to the tsetse immune responses and that the humoral immune system does not play a significant role in controlling *S. glossinidius* proliferation. Our results are in agreement with studies on natural *Drosophila*-facultative endosymbiont associations, including *Spiroplasma* and *Wolbachia*, where no impact on the immune gene expression of their native hosts was observed (Herren and Lemaitre, 2011; Rances et al., 2013; Chrostek et al., 2014). *Spiroplasma* bacteria were neither detected nor affected by the *D. melanogaster* immune system, but their proliferation was shown to be constrained by the availability of hemolymph lipids which correlated to the nutritional state of the host (Herren et al., 2014). This mechanism could also be important for controlling the proliferation of *S. glossinidius*. Indeed, genome sequencing has indicated that endosymbiotic bacteria have reduced metabolic capacities and are dependent on their hosts to provide them with compounds needed for their sustained proliferation (Moran et al., 2008). The *in silico* analysis of the *S. glossinidius* metabolism has already revealed a heavy dependency on carbohydrates for energy production and a complete inactivation of the pathways for L-arginine and thiamine biosynthesis, indicating that *S. glossinidius* is dependent on its host for supply of these metabolites (Belda et al., 2012), which provides an interesting perspective for future research.

The recent discovery of a close non-insect associated relative of *S. glossinidius*, designated *S. praecaptivus*, provided us with the unique opportunity to compare host immune responses toward *S. glossinidius* and an environmental precursor of the *Sodalis*-allied clade of insect symbionts. Indeed, several *Sodalis*-allied symbionts, including *S. glossinidius and S. pierantonius*, have evolved independently from *S. praecaptivus*, as evidenced by comparative genomic analyses, showing that the symbiont genomes are subsets of this free-living relative (Clayton et al., 2012; Oakeson et al., 2014). Exposure of tsetse flies to *S. praecaptivus* resulted in the upregulation of *PGRP-LC* followed by activation of the Imd-pathway along with the induction of genes encoding AMPs while *S. glossinidius* failed to be recognized, indicating that the latter has developed mechanisms to overcome or evade the tsetse immune response during its transition toward symbiosis. Indeed, *S. glossinidius* has been shown *in vitro* to display a high level of resistance against the bactericidal actions of the insect microbicidal peptide diptericin (Hao et al., 2001) and attacin (Hu and Aksoy, 2005). In further support of this data, we showed in a previous study that *S. glossinidius* utilizes a PhoP-PhoQ two-component regulatory system to modulate the expression of genes involved in lipid A modifications that confer bacterial resistance to host derived AMPs *in vivo* (Pontes et al., 2011). Biofilm formation has also been suggested as a mechanism for *S. glossinidius* to evade its host immune system (Maltz et al., 2012).

It is interestingly to note that injection of *S. pierantonius* in the weevil’s hemolymph does elicit a potent systemic immune response (Anselme et al., 2008). These data indicate that, when present in the hemolymph, *S. pierantonius* is recognized by the immune system as an intruder and attests that the endosymbiont is tolerated in the bacteriocyte cells only. This PGRP-LC mediated systemic immune response has recently been shown to be triggered by diaminopimelic acid-type (DAP)-type peptidoglycan synthesized by *S. pierantonius* (Maire et al., 2019). Indeed, MAMPs such as peptidoglycan are capable of activating a host immune response through interaction with host PRRs. It is noteworthy that although these immune eliciting elements were shown to be absent from the genomes of most long lasting insect endosymbionts (McCutcheon and Moran, 2011), in both *S. pierantonius* and *S. glossinidius* all genes involved in peptidoglycan and lipopolysaccharide (LPS) biosynthesis are preserved (Oakeson et al., 2014). However, analysis of the *S. glossinidius* genome sequence indicates that several immunogenic components of its cell membrane are altered, including a truncated LPS lacking the immunodominant O-antigen and a modified outer-membrane protein A (OmpA) (Toh et al., 2006). These *S. glossinidius*-specific polymorphisms in OmpA have been linked to host tolerance to this bacterium (Weiss et al., 2008) and may explain in part why it does not trigger the immune response of its host although abundantly present in the extracellular environment.

Finally, we report on the impact of *S. glossinidius* on the tsetse fly’s vector competence for major trypanosome parasites, i.e., *T. brucei* sp. and *T. congolense*. Although its actual role in the ability of tsetse flies to acquire and transmit the parasite still remains controversial, *S. glossinidius* has been suggested to be associated with an increased competence of tsetse for trypanosome transmission (Dale and Welburn, 2001). However, to date no appropriate experimental model has been available to verify this hypothesis. In our study, the trypanosome infection rates in *Gmm*^*Sod+*^ and *Gmm*^*Sod*^***^–^*** flies did not demonstrate significant differences in midgut establishment nor at the final developmental stage in the salivary glands (*T. brucei*) or proboscis (*T. congolense*). Moreover, no differences were observed in parasite abundance of the midgut infections in *Gmm*^*Sod*+^ and *Gmm*^*Sod*^***^–^*** flies. While these experiments clearly show that the mere presence of *S. glossinidius* does not impact tsetse’s vector competence, they cannot exclude a more subtle effect. Indeed, if *S. glossinidius* poses a metabolic cost on its host, it could indirectly impact the growth and/or development of the parasite within the tsetse fly. Recently it was shown that the endosymbiont *Spiroplasma* protects *Drosophila* against parasitoid wasps by depleting its host resources that the pathogen normally relies on (Paredes et al., 2016). This effect was more conspicuous under conditions of host nutrient limitation, which is also known to be an important stressor for natural tsetse fly populations. Ongoing metabolomic experiments in *Gmm*^*Sod*+^ and *Gmm*^*Sod*^***^–^*** flies under normal and nutrient limiting conditions will provide more information about the transfer of metabolites between the tsetse host and the *S. glossinidius* endosymbiont and its relevance in the context of the fly vector competence for African trypanosomes.

In conclusion, the results from our study suggest that not only host immune tolerance but also bacterial immune evasion are involved in the establishment and maintenance of the *S. glossinidius*−tsetse symbiotic association. This work provides new opportunities to study the biological impact of *S. glossinidius* on the tsetse fly’s physiology and paves the way to finally unravel the functional role of this symbiosis in tsetse flies.

## Data Availability

The raw sequencing reads have been deposited at the Short Read Archive (http://www.ncbi.nlm.nih.gov/sra) in the BioProject with the Accession Number PRJNA476840.

## Ethics Statement

Breeding and experimental work with tsetse flies was approved by the Scientific Institute Public Health department Biosafety and Biotechnology (SBB 219.2007/1410). Animal ethics approval for the tsetse fly feeding on live animals was obtained from the Animal Ethical Committee of the Institute of Tropical Medicine Antwerp (Ethical clearance No. BM2012-6). The experiments, and maintenance and care of animals complied with the guidelines of the European Convention for the Protection of Vertebrate Animals used for Experimental and other Scientific Purposes (CETS No. 123).

## Author Contributions

KT, LD, and JV designed the experiments. KT and LD performed the experiments and analyzed the data. IM and KT performed the bioinformatic analysis. KT, LD, IM, and JV wrote the manuscript. All authors have read and approved the final manuscript.

## Conflict of Interest Statement

The authors declare that the research was conducted in the absence of any commercial or financial relationships that could be construed as a potential conflict of interest.

## Abbreviations

AMP: antimicrobial peptide
DEG: differentially expressed gene
dif: dorsal-related immunity factor
GNBP1: gram-negative binding protein 1
iap2: inhibitor of apoptosis 2
Imd: immune deficiency
JAK/STAT: janus kinase/signal transduction and activator of transcription
key: kenny
PGRP: peptidoglycan recognition protein
PM: peritrophic matrix
SOCS: suppressor of cytokine signaling
STAT92E: signal transducer and activator of transcription 92E
USP36: ubiquitin-proteasome related protein 36
vir-1: virus-induced RNA 1.
