# Supplementary material for: The Tsetse Fly Displays an Attenuated Immune Response to Its Secondary Symbiont, Sodalis glossinidius
Source: Front Microbiol. 2019 Jul 24;10:1650. doi: 10.3389/fmicb.2019.01650 (PMC6668328; doi:10.3389/fmicb.2019.01650)
Supplement: Supplementary file 1 [file Table_1.DOCX]

**Additional file 1. Impact of the different streptozotocin treatments on the tsetse fly viability and fecundity.** The table accompanies Additional file 2. G: gonotrophic cycle, NA: not applicable.

| Administration method | Streptozotocin dose | No. flies | Tsetse viability | Tsetse fecundity |
| --- | --- | --- | --- | --- |
| Injection | 20 µg | 20 | Lethal | NA |
| Injection and 3 blood meals | 20 µg + 20 µg/ml | 20 | Lethal | NA |
| Injection | 0.25 µg -5 µg | 90 | Not-lethal | NA |
| Injection | 0.5 µg | 15 | Not-lethal | NA |
| Injection and 3 blood meals | 0.5 µg + 20 µg/ml | 15 | Not-lethal | NA |
| 3 blood meals | 20 µg/ml | 15 | Not-lethal | NA |
| Injection and 6 blood meals | 0.5 µg + 20 µg/ml | 30 | Not-lethal | No larvae |
| Injection and 3 blood meals | 0.5 µg + 20 µg/ml | 60 | Not-lethal | No larvae |
| Injection and 3 blood meals | 0.25 µg + 10 µg/ml | 50 | Not-lethal | G_1_, G_2_ |
| 3 blood meals | 10 µg/ml | 50 | Not-lethal | G_1_, G_2_ |
| 3 blood meals | 0.5 µg/ml -10 µg/ml | 80 | Not-lethal | NA |
| Continuous blood meals | 10-15 µg/ml | 100 | Not-lethal | No G_2_ |
| Continuous blood meals | 2.5-5 µg/ml | 180 | Not-lethal | No G_2_ |
| Continuous blood meals | 0.5 µg/ml | 90 | Not-lethal | G_1_, G_2_, G_3_, etc. |
