# Supplementary material for: The Tsetse Fly Displays an Attenuated Immune Response to Its Secondary Symbiont, Sodalis glossinidius
Source: Front Microbiol. 2019 Jul 24;10:1650. doi: 10.3389/fmicb.2019.01650 (PMC6668328; doi:10.3389/fmicb.2019.01650)
Supplement: Supplementary file 2 [file Table_2.DOCX]

**Additional file 3. Primer sequences used for qRT-PCR expression analysis.** ^1^Genes used for transcriptome validation of the RNA-seq analysis. ^2^Genes used for the *S. glossinidius*-concentration, *S. praecaptivus*, and bacterial exposure in *S. glossinidius*+/- flies experiments; dif: dorsal-related immunity factor; dnr1: defense repressor 1; GNBP1: Gram-negative binding protein 1; iap2: inhibitor of apoptosis 2; PGRP: peptidoglycan recognition protein; SOCS: suppressor of cytokine signaling; vir-1: virus-induced RNA 1.

| Gene name | Gene ID | Primer efficiency | Amplicon  (bp) | Primer Fwd | Primer Rev |
| --- | --- | --- | --- | --- | --- |
| *PGRP-LC^2^* | **GMOY006094** | 0.97 | 90 | GGCGCCCACACAAAAGGATA | CAATTGCGCATCGTTCGGTA |
| *PGRP-LB^1,2^* | **GMOY006730** | 0.95 | 102 | GAAACGACGCCACTGCATAG | GATCACAAACGCAGAAGGGC |
| *GNBP1^1^* | **GMOY011181** | 0.94 | 112 | CAAATGATGCGTGGGGGTTC | GGGCTGATACGGTTGCGATA |
| *relish^1,2^* | **GMOY013090** | 1.04 | 116 | AGGGGGTCGCGAATTGACTA | CCACCCGGAACGCTTCATAG |
| *iap2^1^* | **GMOY003276** | 0.90 | 158 | GAAGCACGTTTGCGGTCATT | ACTACGTCACCGTACTCCCA |
| *caspar^1^* | **GMOY005909** | 0.94 | 118 | ACAGCCAGAAGGTAATCGCC | CCCCGTCCACTCTTGATGTC |
| *dnr1^1^* | **GMOY000299** | 0.93 | 135 | AGTTCGCGGCGTTGTAACTA | TGATCGTGTACCTCCCGACT |
| *dorsal^1^* | **GMOY004477** | 0.90 | 103 | ATCGTTTTCAGCCATCCGGT | GGTGGGAGAGGTTGCGTAAA |
| *dif^1^* | **GMOY004479** | 0.94 | 117 | AGGTTCTATACCCGGCGCTA | GGCGCATCTTTTGTAACGCA |
| *Cactus* | **GMOY007166** | 0.91 | 114 | ACGGAGAGTGCAAGTAACGA | ATCTGCTGTTTGGGTGCCAT |
| *SOCS^1^* | **GMOY007838** | 0.98 | 113 | ACAAGACAGTCCAACGGGTC | CGCTGTTGCTTGATTGCTGT |
| *vir-1^1^* | **GMOY003759** | 0.93 | 139 | CTTGGCGTTGAATTTTGCTTTG | GGAATGTATAACCGACACCCG |
| *attacinB* | **GMOY010523** | 1.00 | 182 | TATGGTCATCGCCAAACCGT | TCCTTTCCGTTTTATTTTCCATTCT |
| *attacinD* | **GMOY010524** | 0.95 | 150 | AGCATTTTGGCGGACCGTT | AGGGTTCCGTTGGCACAAATA |
| *defensinA^2^* | **AF368907.1** | 0.97 | 112 | TGTTGCCGTTACTGCTTTGC | GCGTTTCAATTCGCCCGTAA |
| *cecropin^2^* | **GMOY011562** | 0.95 | 89 | GTCAGAGCGAAGCTGGTTGG | TAGACCTTTCACGGTGGCATC |
