# Supplementary material for: The Tsetse Fly Displays an Attenuated Immune Response to Its Secondary Symbiont, Sodalis glossinidius
Source: Front Microbiol. 2019 Jul 24;10:1650. doi: 10.3389/fmicb.2019.01650 (PMC6668328; doi:10.3389/fmicb.2019.01650)
Supplement: Supplementary file 3 [file Table_3.DOCX]

**Additional file 4.**

**Primer sequences used to obtain the complete relish coding sequence.**

| Gene name | Amplicon  (bp) | Primer Fwd | Primer Rev |
| --- | --- | --- | --- |
| *relish* | 2,599 | TGCTTCGGGTTATTCGCCAG | AAGCCAGTTGAGGACACCTTC |

**Primer sequences used to generate the template for in vitro (IT) transcription.**

| Gene name | Amplicon  (bp) | Primer Fwd | Primer Rev |
| --- | --- | --- | --- |
| *IT template* | 479 | TAATACGACTCACTATAGG  GGCCCGATTGGAAATTACTGA | TAATACGACTCACTATAGGGGTCA  TAGCAAAGTCGCACCTG |
