# Supplementary material for: The Tsetse Fly Displays an Attenuated Immune Response to Its Secondary Symbiont, Sodalis glossinidius
Source: Front Microbiol. 2019 Jul 24;10:1650. doi: 10.3389/fmicb.2019.01650 (PMC6668328; doi:10.3389/fmicb.2019.01650)
Supplement: Supplementary file 4 [file Table_4.DOCX]

**Additional file 5**. **Species-specific primer sequences used for qRT-PCR based *in vivo* measurement of the bacterial densities in the tsetse fly.**

| Species | Gene name | Amplicon  (bp) | Primer Fwd | Primer Rev |
| --- | --- | --- | --- | --- |
| *Sodalis glossinidius* | *Exochitinase* | 120 | TGGGGACAGTACGATGGCAGAGC | TCATAGGCGGTCGGGGATAATTGCG |
| *Wigglesworthia glossinidia* | *Thiamine biosynthesis protein* | 130 | AAGTTATGATAGAAGGACCAGGAC | CCCGGAGCAATATCAGTAGTTAG |
| *Wolbachia* sp. | *16S rRNA* | 246 | CATACCTATTCGAAGGGATAG | GGATTAGCTTAGCCTCGC |
| *Glossina morsitans* | *α-tubulin* | 160 | CAAGGAGGACGCTGCGAATA | CCACCACCGAACGAATGGAA |
