# Supplementary material for: The Tsetse Fly Displays an Attenuated Immune Response to Its Secondary Symbiont, Sodalis glossinidius
Source: Front Microbiol. 2019 Jul 24;10:1650. doi: 10.3389/fmicb.2019.01650 (PMC6668328; doi:10.3389/fmicb.2019.01650)
Supplement: Supplementary file 5 [file Table_5.DOCX]

**Additional file 6. Primer sequences used for qRT-PCR based *in vivo* measurement of the trypanosome densities in the tsetse fly.**

| Gene name | Amplicon  (bp) | Primer Fwd | Primer Rev |
| --- | --- | --- | --- |
| *18S rRNA* | 105 | CGCCAAGCTAATACATGAACCAA | TAATTTCATTCATTCGCTGGACG |
