# Supplementary material for: The Tsetse Fly Displays an Attenuated Immune Response to Its Secondary Symbiont, Sodalis glossinidius
Source: Front Microbiol. 2019 Jul 24;10:1650. doi: 10.3389/fmicb.2019.01650 (PMC6668328; doi:10.3389/fmicb.2019.01650)
Supplement: Supplementary file 6 [file Table_6.docx]

**Additional file 8. Sequencing, mapping, and counting results of 10-day old symbiotic (*Gmm^Sod+^*) and *Sodalis*-free (*Gmm^Sod-^*) tsetse flies.** Total reads as obtained after sequencing, UMR: uniquely mapped reads to the *Glossina morsitans morsitans* reference genome (*GMOY1*), % UMR of total input reads, number of transcripts with non-zero counts, % number transcripts with non-zero counts over total number of *Glossina* transcripts (12,969). PCC: Pearson correlation coefficient between biological replicates transcriptomes.

| Tsetse fly group | Biol. repl. | Total reads (M) | UMR to *Gmm* (M) | % UMR | No. transcripts | % No. transcripts | PCC | Biol. repl. |
| --- | --- | --- | --- | --- | --- | --- | --- | --- |
| *Gmm^Sod+^ flies* | R1 | 124.0 | 106.0 | 85.65% | 10,333 | 79.67% | 72.50%  41.75% | R1R2  R1R3 |
|  | R2 | 202.0 | 177.0 | 87.77% | 10,713 | 82.60% | 83.87%  63.73% | R2R3  R2R4 |
|  | R3 | 127.0 | 107.0 | 84.54% | 10,516 | 81.09% | 46.25%  79.24% | R3R4  R3R5 |
|  | R4 | 113.0 | 85.7 | 75.70% | 10,268 | 79.17% | 90.32%  59.18% | R1R4  R4R5 |
|  | R5 | 108.0 | 98.1 | 90.53% | 10,534 | 81.22% | 72.54%  93.37% | R1R5  R2R5 |
| *Gmm^Sod-^*  *flies* | R1 | 81.0 | 67.6 | 83.47% | 10,166 | 78.39% | 55.13%  75.24% | R1R2  R1R3 |
|  | R2 | 131.0 | 114.0 | 87.34% | 10,470 | 80.73% | 68.04%  54.17% | R2R3  R2R4 |
|  | R3 | 148.0 | 125.0 | 84.18% | 10,710 | 82.58% | 78.02% | R3R4 |
|  | R4 | 145.0 | 124.0 | 85.33% | 10,555 | 81.39% | 90.68% | R1R4 |
