# Supplementary material for: The Tsetse Fly Displays an Attenuated Immune Response to Its Secondary Symbiont, Sodalis glossinidius
Source: Front Microbiol. 2019 Jul 24;10:1650. doi: 10.3389/fmicb.2019.01650 (PMC6668328; doi:10.3389/fmicb.2019.01650)
Supplement: Supplementary file 8 [file Table_8.docx]

**Additional file 10. Sequencing, mapping, and counting results of *Sodalis*-free flies exposed to 10^6^ CFU *S. glossinidius* (*Gmm^Sod-/Sod^*^+^), 10^5^ CFU *E. coli* (*Gmm^Sod-/Ecoli^*^+^), or sterile saline (*Gmm^Sod^*^-/saline^).** Total reads as obtained after sequencing. UMR: uniquely mapped reads to the *Glossina morsitans morsitans* reference genome (*GMOY1*), % UMR of total input reads, number of transcripts with non-zero counts, % number transcripts with non-zero counts over total number of *Glossina* transcripts (12,969). PCC: Pearson correlation coefficient between biological replicates transcriptomes.

| Tsetse fly group | Biol. repl. | Total reads (M) | UMR to *Gmm* (M) | % UMR | No. transcripts | % No. transcripts | PCC | Biol. repl. |
| --- | --- | --- | --- | --- | --- | --- | --- | --- |
| *Gmm^Sod-/Sod+^* | R1 | 112.0 | 97.2 | 86.58% | 10,550 | 81.35% | 88.49% | R1R2 |
|  | R2 | 93.6 | 84.4 | 90.15% | 10,694 | 82.46% | 90.28% | R1R3 |
|  | R3 | 81.6 | 73.6 | 90.06% | 10,434 | 80.46% | 72.85% | R2R3 |
| *Gmm^Sod-/Ecoli+^* | R1 | 84.6 | 76.4 | 90.16% | 10,554 | 81.38% | 77.35% | R1R2 |
|  | R2 | 82.0 | 71.8 | 87.51% | 10,517 | 81.10% | 79.74% | R1R3 |
|  | R3 | 112.0 | 99.0 | 88.59% | 10,582 | 81.60% | 97.85% | R2R3 |
| *Gmm^Sod^*^-/saline^ | R1 | 83.4 | 74.4 | 89.26% | 10,413 | 80.30% | 95.83% | R1R2 |
|  | R2 | 79.6 | 70.0 | 87.85% | 10,356 | 79.86% | 97.88% | R1R3 |
|  | R3 | 76.6 | 68.8 | 89.81% | 10,409 | 80.27% | 98.67% | R2R3 |
