# Supplementary material for: The Tsetse Fly Displays an Attenuated Immune Response to Its Secondary Symbiont, Sodalis glossinidius
Source: Front Microbiol. 2019 Jul 24;10:1650. doi: 10.3389/fmicb.2019.01650 (PMC6668328; doi:10.3389/fmicb.2019.01650)
Supplement: Supplementary file 9 [file Table_9.DOCX]

**Additional file 11.** **Log_2_ fold changes in expression obtained by RNA-seq and qRT-PCR used for transcriptome validation of the RNA-seq data.**

| Gene | Gene ID | RNA-seq  log_2_ fold change | qRT-PCR log_2_fold change |
| --- | --- | --- | --- |
| *PGRP-LB* | **GMOY006730** | 1.53 | 1.76 |
| *GNBP1* | **GMOY011181** | 1.07 | 1.24 |
| *dnr1* | **GMOY000299** | -0.36 | -0.59 |
| *caspar* | **GMOY005909** | 0.49 | 0.43 |
| *iap2* | **GMOY003276** | 1.27 | 1.36 |
| *vir-1* | **GMOY003759** | -0.05 | -0.08 |
| *dorsal* | **GMOY004477** | 0.70 | 0.83 |
| *relish* | **GMOY013090** | 1.68 | 2.04 |
| *dif* | **GMOY004479** | 0.00 | 0.30 |
| *SOCS* | **GMOY007838** | 2.61 | 3.45 |
| PCC: 98.98% | | | |
