# Supplementary figures and images for: The Tsetse Fly Displays an Attenuated Immune Response to Its Secondary Symbiont, Sodalis glossinidius
Source: Front Microbiol. 2019 Jul 24;10:1650. doi: 10.3389/fmicb.2019.01650 (PMC6668328; doi:10.3389/fmicb.2019.01650)

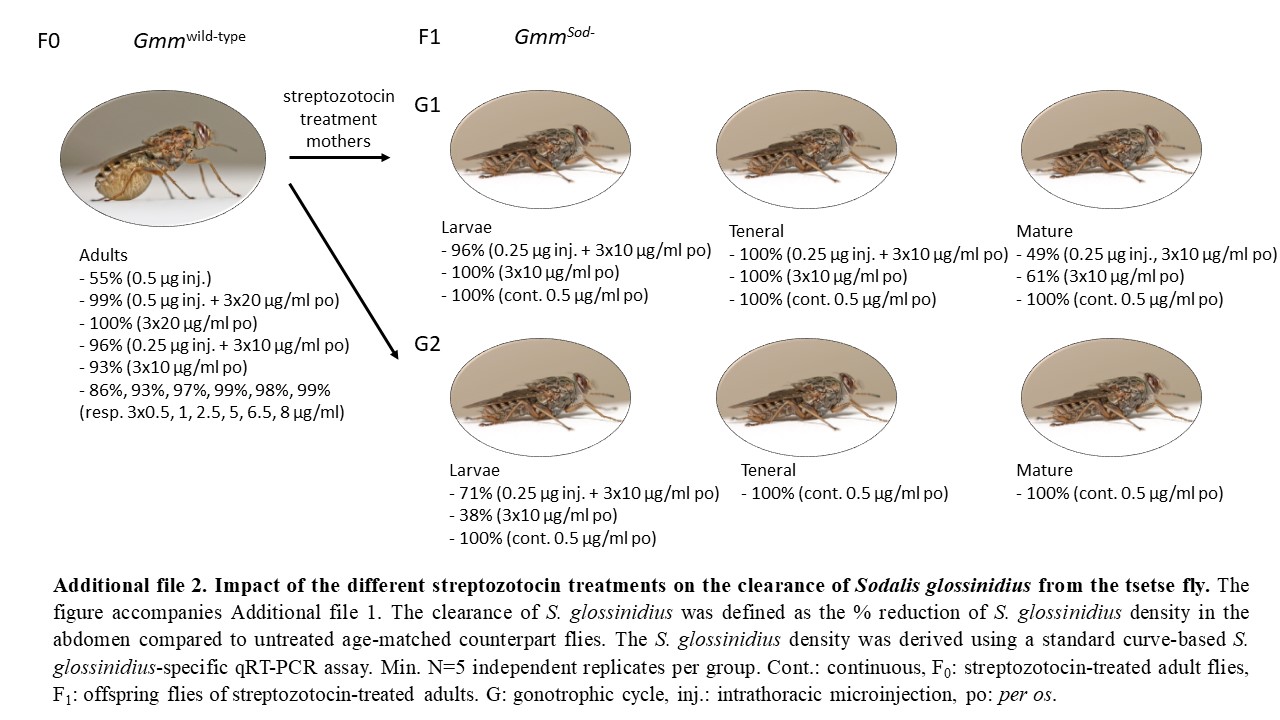

Supplement: Supplementary file 10 [file Image_1.JPEG]

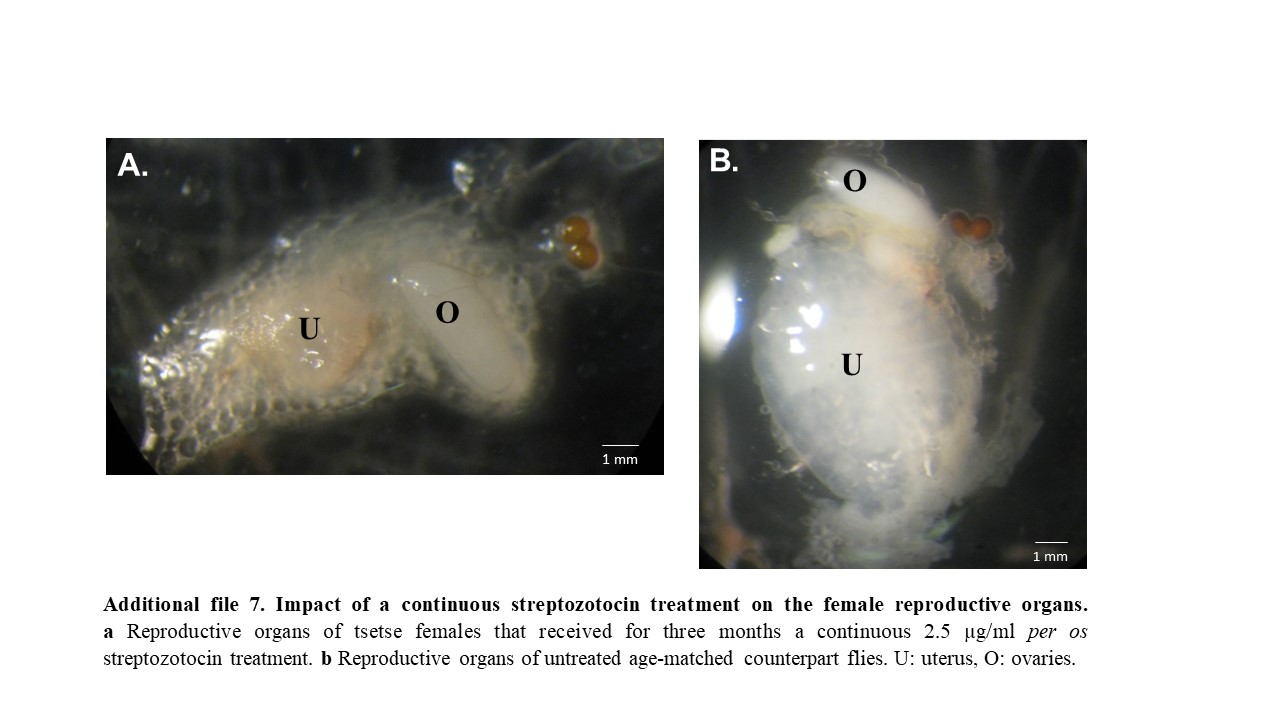

Supplement: Supplementary file 11 [file Image_2.JPEG]

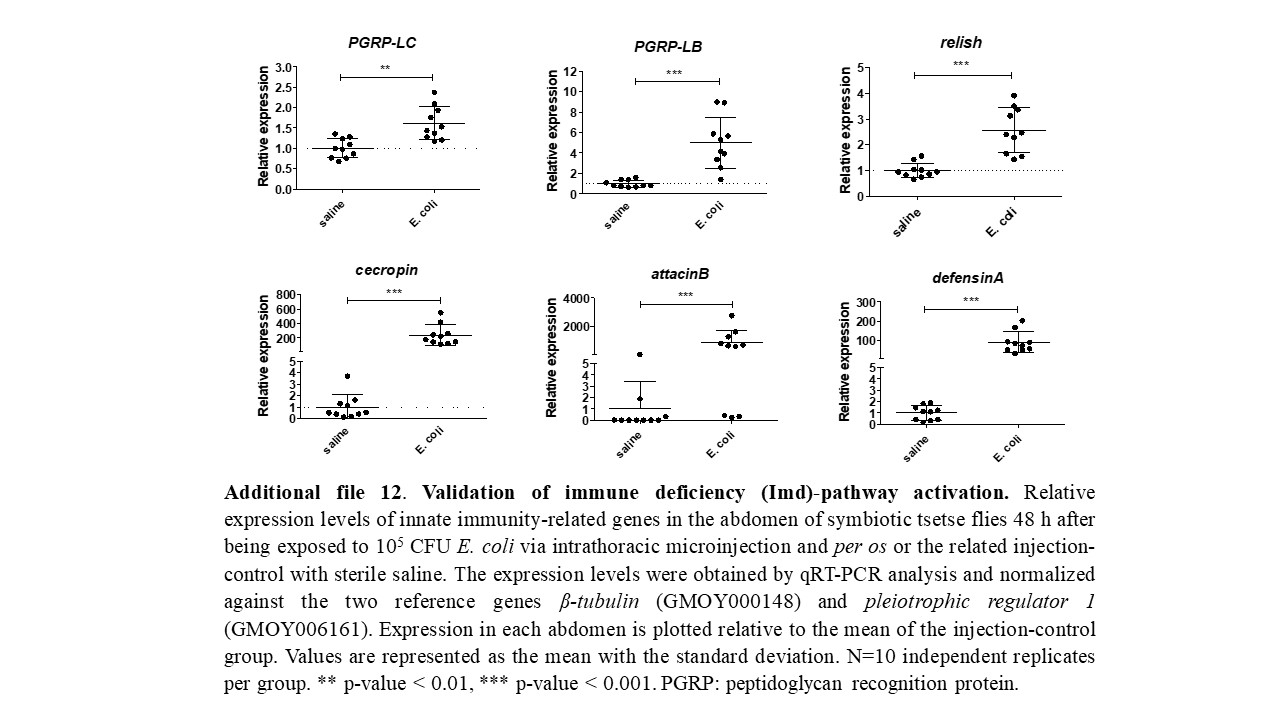

Supplement: Supplementary file 12 [file Image_3.JPEG]

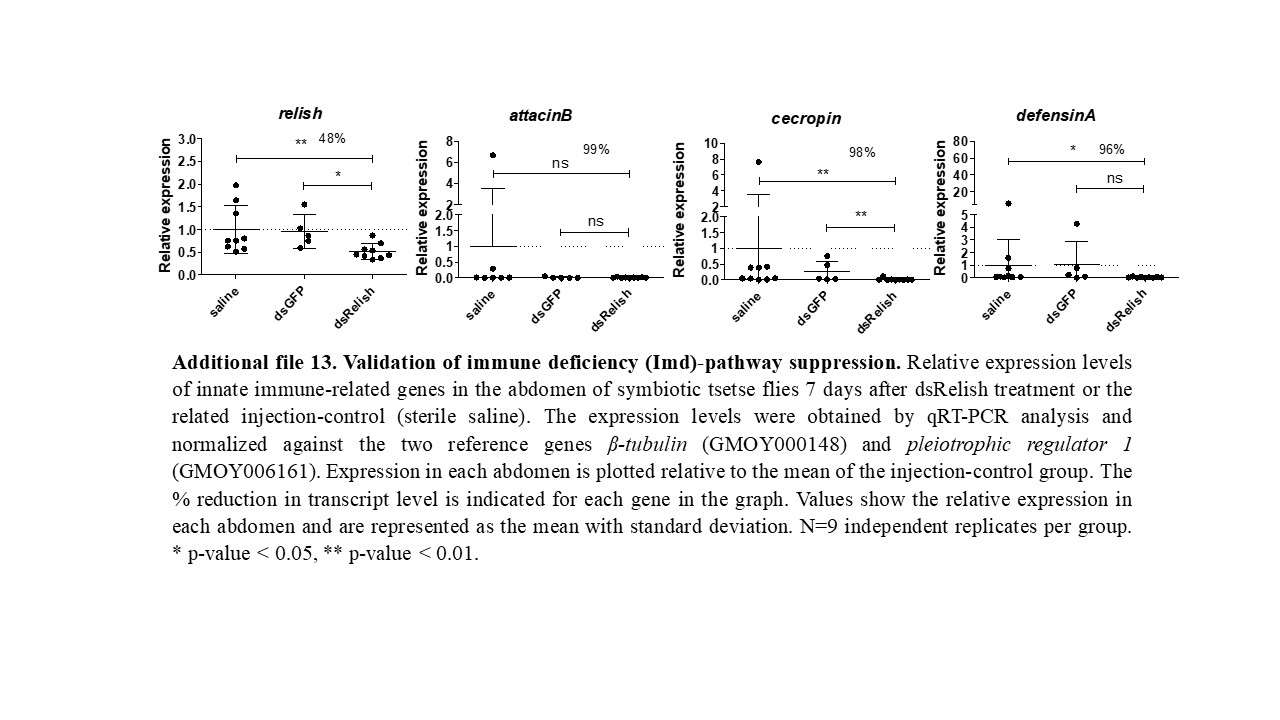

Supplement: Supplementary file 13 [file Image_4.JPEG]
